# Cell-type-specific PtrWOX4a and PtrVCS2 form a regulatory nexus with a histone modification system for stem cambium development in *Populus trichocarpa*

**DOI:** 10.1038/s41477-022-01315-7

**Published:** 2023-01-09

**Authors:** Xiufang Dai, Rui Zhai, Jiaojiao Lin, Zhifeng Wang, Dekai Meng, Meng Li, Yuli Mao, Boyuan Gao, Hongyan Ma, Baofeng Zhang, Yi Sun, Shuang Li, Chenguang Zhou, Ying-Chung Jimmy Lin, Jack P. Wang, Vincent L. Chiang, Wei Li

**Affiliations:** 1 State Key Laboratory of Tree Genetics and Breeding, Northeast Forestry University, Harbin, China; 2 National Key Laboratory of Plant Molecular Genetics and CAS Center for Excellence in Molecular Plant Sciences, Chinese Academy of Sciences, Shanghai, China; 3 Department of Life Sciences and Institute of Plant Biology, College of Life Science, National Taiwan University, Taipei, Taiwan China; 4 Forest Biotechnology Group, Department of Forestry and Environmental Resources, North Carolina State University, Raleigh, NC USA

**Keywords:** Plant molecular biology, Plant stem cell

## Abstract

Stem vascular cambium cells in forest trees produce wood for materials and energy. WOX4 affects the proliferation of such cells in *Populus*. Here we show that Ptr*WOX4a* is the most highly expressed stem vascular-cambium-specific (VCS) gene in *P. trichocarpa*, and its expression is controlled by the product of the second most highly expressed VCS gene, Ptr*VCS2*, encoding a zinc finger protein. PtrVCS2 binds to the Ptr*WOX4a* promoter as part of a PtrWOX13a–PtrVCS2–PtrGCN5-1–PtrADA2b-3 protein tetramer. PtrVCS2 prevented the interaction between PtrGCN5-1 and PtrADA2b-3, resulting in H3K9, H3K14 and H3K27 hypoacetylation at the Ptr*WOX4a* promoter, which led to fewer cambium cell layers. These effects on cambium cell proliferation were consistent across more than 20 sets of transgenic lines overexpressing individual genes, gene-edited mutants and RNA interference lines in *P. trichocarpa*. We propose that the tetramer–Ptr*WOX4a* system may coordinate genetic and epigenetic regulation to maintain normal vascular cambium development for wood formation.

## Abstract (web summary)

This study revealed a regulatory system in which transcription factor and histone acetylation functions are coordinated for maintaining normal stem vascular cambium development for wood formation in *Populus trichocarpa*.

## Main

Forest tree species are the best systems to study wood formation because they perennially produce abundant wood through lateral growth. In the stem vascular meristem of forest trees, the fusiform initials (or stem cells) self-renew and differentiate into vessels, fibres and rays to increase stem diameter^1,2^ and form wood. Fusiform initials are the only cells able to produce derivatives toward both the xylem and the phloem^1–4^. The wood-cell lineage begins with the division of the fusiform initial, which is believed to be located immediately below a large phloem cell^1,5,6^.

**Fig. 1:**
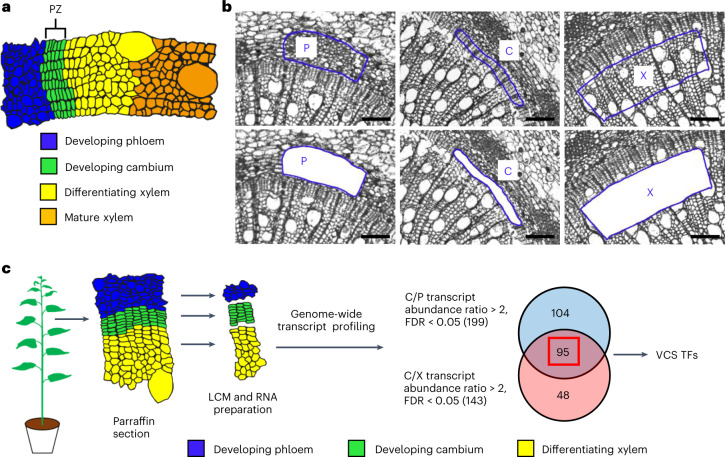
Identification of VCS TFs in *P. trichocarpa*. **a**, The arrangement of vascular developing phloem (blue), developing cambium (green), differentiating xylem (yellow) and mature xylem (orange) in a stem cross-section of *P. trichocarpa*. PZ, proliferation zone. **b**, The developing phloem (P), developing cambium (C) and differentiating xylem (X) cells can be readily captured by LCM. Representative images from one biological replicate are shown. Cells for the other two biological replicates were prepared in the same way. The blue outlines represent regions excised and collected by LCM. Scale bars, 100 µm. **c**, Schematic workflow for the identification of VCS TF genes. The 8th internode from the 4-month-old *P. trichocarpa* stems were used for paraffin section. Three cell types were collected by LCM, and the total RNA was extracted for genome-wide transcript profiling. A total of 199 TFs were identified with a C/P transcript abundance ratio > 2 (FDR < 0.05), and 143 TFs were identified with a C/X transcript abundance ratio > 2 (FDR < 0.05). The overlap between the 199 TFs and the 143 TFs was 95 TFs, which were identified as VCS TFs.

Sanio demonstrated in 1873^7^ that in the stem vascular meristem of Scots pine (*Pinus sylvestris*), a fusiform initial renews and divides in the proliferation zone (the green cell area in Fig. 1a) into roughly eight vascular cambium cell layers before these cells differentiate into vessels and fibres to make wood. The presence of the proliferation zone, with a fixed number of cambium cell layers, has been widely confirmed in gymnosperms^3,5,8,9^ and angiosperm trees including *Populus*^6,10–14^. This progression of fusiform initial development is analogous to that in shoot apical meristems (SAMs) and root apical meristems (RAMs), where the stem cells allow plants to elongate axially.

Knowledge of SAMs and RAMs derived from *Arabidopsis* is substantial^15^. *Arabidopsis* WUSCHEL (WUS)^16^ and WUS-related HOMEOBOX5 (WOX5)^17^ each organize a feedback regulatory loop to modulate stem cell homeostasis in the SAM^18^ and the RAM^19^, respectively. At a higher regulatory level, histone acetylation^20,21^ and deacetylation^22^ of *WUS* in the chromatin activates and suppresses *WUS* expression, respectively, to determine floral meristem activity. Histone trimethylation modifications of the *WOX5* promoter regulate its gene expression to affect RAM development in *Arabidopsis* roots^23^.

WOX4 is a common regulator of hypocotyl procambium proliferation^24^ and root cambium development^25^ in *Arabidopsis*. In stems, *WOX4* expression is induced in a *WOX4*-centred regulatory signalling pathway to promote cambium cell proliferation^24,26,27^. In *Arabidopsis* roots, 32 cambium transcription factors (TFs) have been identified, of which 13 are interconnected through predicted direct interactions to form a layered network^25^. In this network, *WOX4* is a major node in regulating vascular cambium development^25^. Epigenetic control of *WOX4* expression has not yet been reported.

WOX4 is also an important regulator of vascular cambium development in wood formation, but the underlying regulatory system is still in an earlier stage of identification^28,29^. A basic knowledge of all key stem vascular-cambium-specific (VCS) TF genes is lacking. Ten *Populus* stem-cambium-expressed (specific or non-specific) TF genes—Ptt*WOX4a/b*^30^, Ptr*HB4* (ref. 31), Ptr*HB7* (ref. 32), *PRE* (*popREVOLUTA*)^33^, Ptr*VCM1* and Ptr*VCM2* (ref. 34), *ARK2* (ref. 35), *POPCORONA*^36^ and Pto*TCP20* (ref. 37)—have been reported, and their genetic functions were tested mostly in heterologous *Populus* species. These studies showed the effects of perturbing these TF genes on cambium development but found no clear clues to their underlying regulatory pathways and mechanisms.

In this study, we identified 95 VCS TFs in *P. trichocarpa* stems. We report a regulatory pathway in which the second most abundant *VCS*, Ptr*VCS2*, controls the expression of the most abundant *VCS*, Ptr*WOX4a*, through the system’s epigenetic modification apparatus to regulate the number of cambium cell layers for wood formation.

## Results

### Cell-type transcriptome analysis identified 95 VCS TF genes

We used *P. trichocarpa* as a model wood-forming system to study cambium development. Using laser capture microdissection (LCM), we identified 95 VCS TF genes (Fig. 1a–c, Supplementary Table 1 and Supplementary Text). These genes were numbered from Ptr*VCS1* to Ptr*VCS95* (Supplementary Table 1) on the basis of their transcript levels in the vascular cambium. Ptr*VCS1* (Potri.014G025300, the most abundant *VCS*) is identical to Ptr*WOX4a*^30^, and Ptr*VCS2* (Potri.004G126600, the second most abundant *VCS*) encodes a zinc finger (ZF) protein belonging to a subfamily^38–40^ of ZF-homeodomain (HD) TF proteins^41^. We focused on these two most abundantly expressed *VCS*s and their homologues (Ptr*WOX4a*, Ptr*WOX4b*, Ptr*VCS2* and Ptr*VCS2-h*, described below) to explore the regulatory mechanism behind vascular cambium development in wood formation. Because *RNAi* Ptt*WOX4a* phenotypes were reported previously^30^, we first characterized Ptr*VCS2*.

### Ptr*VCS2* negatively regulates vascular cambium proliferation

**Fig. 2:**
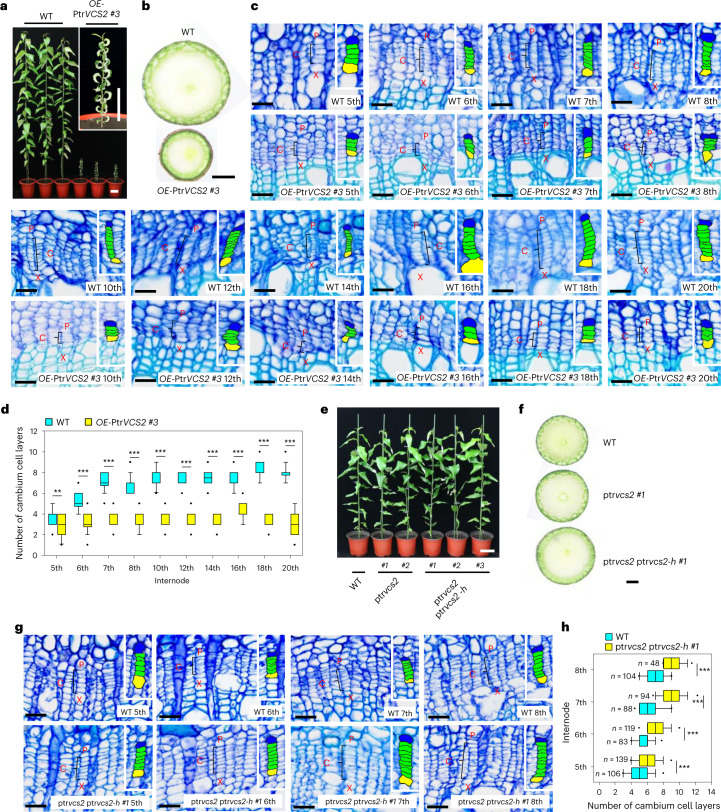
Ptr*VCS2* regulates vascular cambium proliferation in *P. trichocarpa*. **a**, Phenotypes of the WT and *OE*-Ptr*VCS2 #3* transgenics. The inset shows a magnification of an *OE*-Ptr*VCS2 #3* transgenic plant. Scale bars, 10 cm. **b**, Basal stems of the WT and *OE-*Ptr*VCS2 #3* transgenics. Scale bar, 1 mm. **c**, Histochemistry and histological analysis of the WT and *OE*-Ptr*VCS2 #3* transgenics. **d**, Number of cambium cell layers in stem vascular tissues of the WT and *OE*-Ptr*VCS2 #3* transgenics. **e**, Phenotypes of the WT and the ptr*vcs2* and ptr*vcs2* ptr*vcs2-h* mutants. Scale bar, 10 cm. **f**, Basal stems of the WT and the ptr*vcs2 #1* and ptr*vcs2* ptr*vcs2-h #1* mutants. Scale bar, 1 mm. **g**, Histochemistry and histological analysis of the WT and ptr*vcs2* ptr*vcs2-h #1* mutants. **h**, Number of cambium cell layers in stem vascular tissues of the WT and ptr*vcs2* ptr*vcs2-h #1* mutants. In **c** and **g**, the cross-sections were stained with toluidine blue O. Scale bars, 25 µm. The black brackets mark the cambium cells in one radial cell file. The insets show close-ups of cambium cells (green), adjacent phloem cells (blue) and adjacent xylem cells (yellow). In **d** and **h**, the number of cambium cell layers of at least ten radial cell files was counted within one cross-section from each biological replicate. Three biological replicates were analysed. *n* = 30 for **d**; *n* for **h** is shown in the panel. The boxes show the median and the upper and lower quantiles, and the whiskers represent the data range excluding outliers. Two-tailed Student’s *t*-test: **P < 0.01; ***P < 0.001. The *P* values versus the WT control for the *OE*-Ptr*VCS2 #3* transgenics in **d** are as follows: 5th, 0.0025; 6th, <0.0001; 7th, <0.0001; 8th, <0.0001; 10th, <0.0001; 12th, <0.0001; 14th, <0.0001; 16th, <0.0001; 18th, <0.0001; 20th, <0.0001. The *P* values versus the WT control for the ptr*vcs2* ptr*vcs2-h #1* mutants in **h** are as follows: 5th, <0.0001; 6th, <0.0001; 7th, <0.0001; 8th, <0.0001. Source data

**Extended Data Fig. 1:**
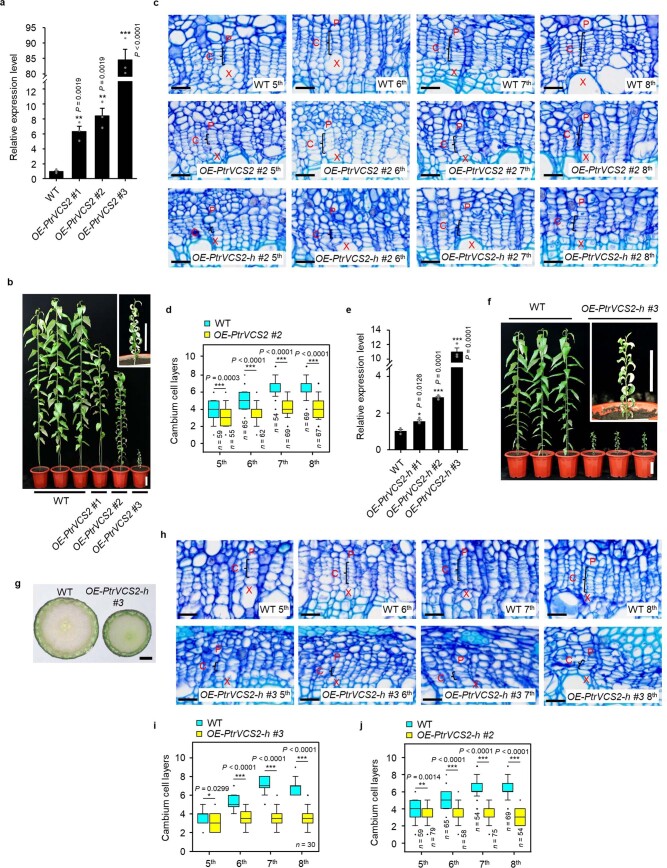
Overexpression of *PtrVCS2* and *PtrVCS2-h* in *P. trichocarpa* and cambium phenotype analysis. **a**, Relative expression levels of *PtrVCS2* in wild-type (WT) and *OE-PtrVCS2* transgenic plants, as determined by RT-qPCR. **b**, Phenotypes of WT and *OE*-*PtrVCS2* transgenics. The inset shows the magnification of an *OE*-*PtrVCS2 #3* transgenic plant. Scale bars, 10 cm. **c**, Histochemistry and histological analysis of WT, *OE-PtrVCS2 #2*, and *OE-PtrVCS2-h #2* transgenics. **d**, Number of cambium cell layers in stem vascular tissues of WT and *OE*-*PtrVCS2* #2 transgenics. **e**, Relative expression levels of *PtrVCS2-h* in WT and *OE-PtrVCS2-h* transgenic plants as determined by RT-qPCR. In (**a**) and (**e**), data are shown as mean±s.e.m.; *n* = 3 biological replicates. Asterisks indicate significant difference between the transgenic and WT plants, *P < 0.05, **P < 0.01, ***P < 0.001 (Two-tailed Student’s *t*-test). **f**, Phenotypes of WT and *OE*-*PtrVCS2-h* #3 transgenics. The inset shows the magnification of an *OE*-*PtrVCS2-h* transgenics plant. Scale bars, 10 cm. **g**, Basal stem of WT and *OE-PtrVCS2-h #3* transgenics. Scale bars, 1 mm. **h**, Histochemistry and histological analysis of WT and *OE*-*PtrVCS2-h* #3 transgenics. In (**c**) and (**h**), cross-sections of the 5th–8th internodes of *P. trichocarpa* stems were stained with toluidine blue O. Black brackets mark the cambium cells in one radial cell file. Scale bars, 25 µm. C, cambium; P, phloem, X, xylem. **i**,**j**, Number of cambium cell layers in stem vascular tissues of WT and *OE*-*PtrVCS2-h* #3 (**i**) and *OE-PtrVCS2-h #2* (**j**) transgenics. In (**d**), (**i**) and (**j**), the number of cambium cell layers of at least ten radial cell files was counted within one cross-section from each biological replicate. Three biological replicates were analyzed. *n* = 30 for (**i**); *n* for (**d**) and (**j**) are shown in the panel. Boxes show the median with the upper and lower quantiles, and the whiskers represent the data range excluding outliers. Two-tailed Student’s *t*-test: *P < 0.05, **P < 0.01, ***P < 0.001. Source data

We generated Ptr*VCS2* overexpression lines and selected two, *OE*-Ptr*VCS2 #2* (Extended Data Fig. 1a,b) and *OE*-Ptr*VCS2 #3* (Fig. 2a,b), that had a high increase in the Ptr*VCS2* transcript level (~8-fold in #2 and ~84-fold in #3) in stem cambium and stunted growth in height and stem diameter (Fig. 2a,b and Extended Data Fig. 1a,b) for further analysis. Stem cross-sections revealed that all *OE*-Ptr*VCS2* internodes examined (5th to 20th from #3 in Fig. 2c,d and 5th to 8th from #2 in Extended Data Fig. 1c,d and Supplementary Text) lacked a fixed number (four to six) of cambium cell layers compared with the wild type (WT). The same abatement of four to six cambium cell layers also occurred in stem internodes of the overexpression lines when compared with those of the WT after the same growth or stem elongation period (30 days; Supplementary Fig. 3). These results suggest inbuilt cell-layer abatements along stem elongation when Ptr*CVS2* transcripts were elevated.

**Extended Data Fig. 2:**
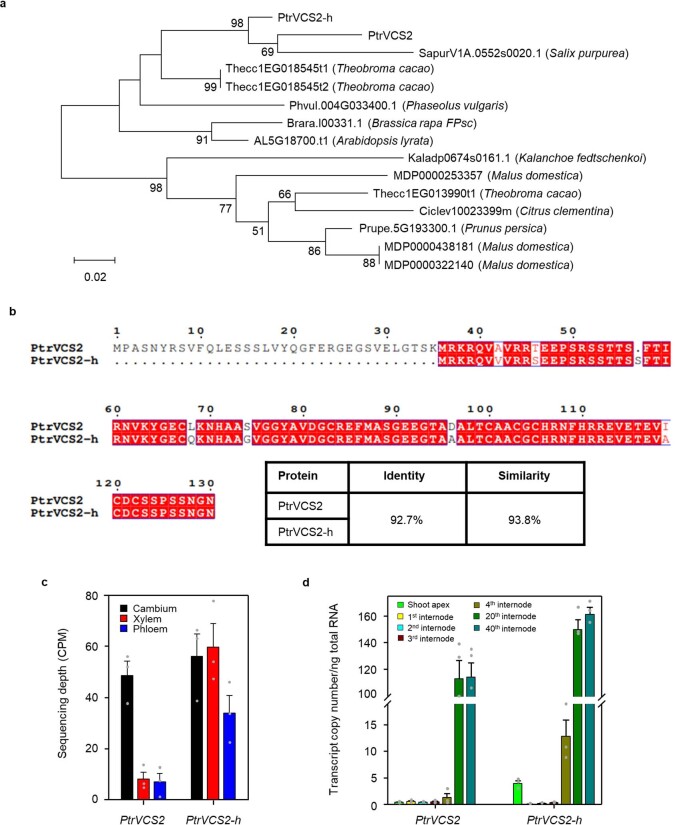
Expression patterns of *PtrVCS2* and its homolog *PtrVCS2-h*. **a**, Phylogenetic tree of PtrVCS2 and homologous proteins from 10 species. The homologous protein sequences are more than 60% similarity with PtrVCS2. The phylogenetic tree was constructed using MEGA 5 with the neighbor-joining method and 1000 bootstrap replicates. Bar, 0.02 changes per amino acid position. **b**, PtrVCS2 and PtrVCS2-h proteins share 92.7% sequence identity and 93.8% sequence similarity. Sequence alignment was carried out with ClustalW. **c**, Transcript abundance of *PtrVCS2* and *PtrVCS2-h* in three cell types. Cambium, xylem and phloem cells in the 8th internode of *P. trichocarpa* stems were collected by LCM for RNA-seq analysis. CPM, counts per million. The data are shown as mean±s.e.m.; *n* = 3 biological replicates. **d**, Transcript abundance of *PtrVCS2* and *PtrVCS2-h*, as determined by RT-qPCR in the shoot apices (containing the apical meristem, leaf primordia, developing leaves and early vascular tissues), 1–4th, 20th and 40th internodes of *P. trichocarpa* stems. The data are shown as mean±s.e.m.; *n* = 3 biological replicates. Source data

**Extended Data Fig. 3:**
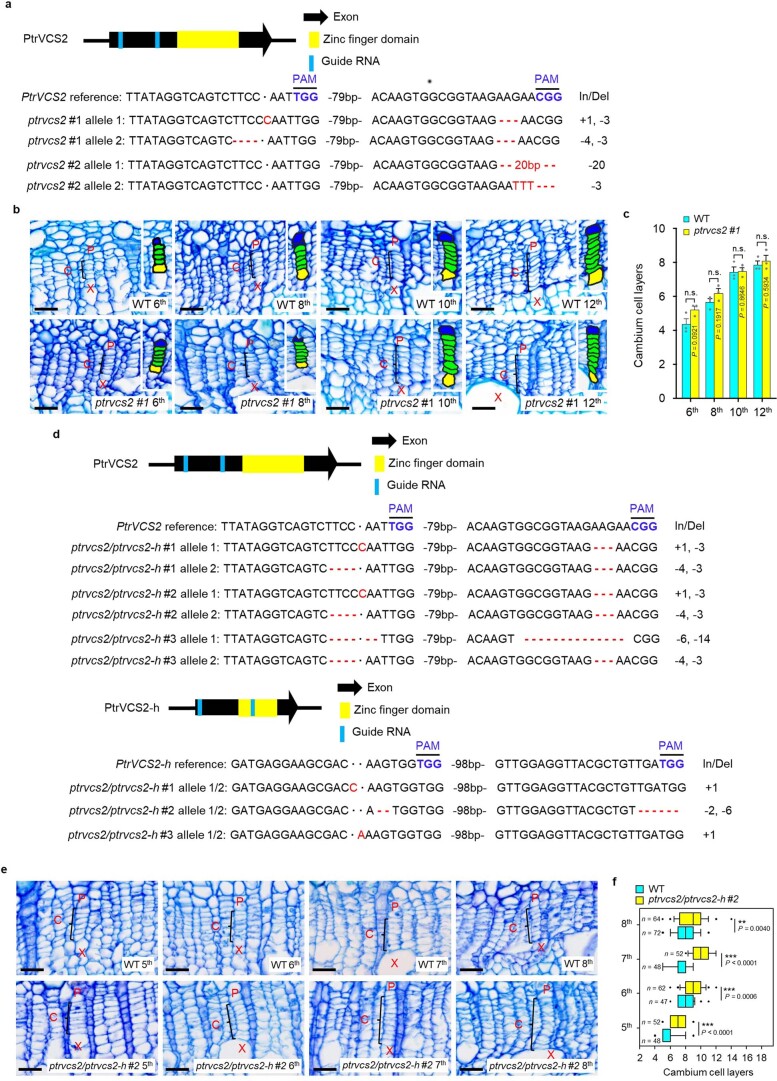
Identification of *ptrvcs2* single mutants and *ptrvcs2/ptrvcs2-h* double mutants and analysis of their cambium phenotypes. **a**,**d**, Mutations at the sgRNA target sites for *PtrVCS2* and *PtrVCS2-h* in two independent *ptrvcs2* mutant lines (**a**) and three independent *ptrvcs2/ptrvcs2-h* mutant lines (**d**). Deleted nucleotides are depicted as red dashes, and substitutions or inserted nucleotides are represented in red font. Blue font indicates the protospacer-adjacent motif (PAM), and the nucleotide length of insertions and/or deletions (In/Del) is presented on the right. **b**, Histochemistry and histological analysis of WT and *ptrvcs2 #1* mutants. Insets show close-ups of the cambium cells (green), adjacent phloem cells (blue) and adjacent xylem cells (yellow). **c**, Number of cambium cell layers in stem vascular tissues of WT and *ptrvcs2 #1* mutant plants. The data are shown as mean±s.e.m.; *n* = 3 biological replicates. The number of cambium cell layers of ten radial cell files was counted within one cross-section from each biological replicate. The average number of cambium cell layers from each biological replicate was analyzed. Two-tailed Student’s *t*-test were used for statistical analysis, and n.s. denotes no significant difference. **e**, Histochemistry and histological analysis of WT and *ptrvcs2/vcs2-h #2* mutants. In (**b**) and (**e**), cross-sections of *P. trichocarpa* stem internodes were stained with toluidine blue O. Black brackets mark the cambium cells in one radial cell file. Scale bars, 25 µm. C, cambium; P, phloem; X, xylem. **f**, Number of cambium cell layers in stem vascular tissues of WT and *ptrvcs2/vcs2-h #2* mutants. Boxes show the median with the upper and lower quantiles, and the whiskers represent the data range excluding outliers. The number of cambium cell layers of at least ten radial cell files was counted within one cross-section from each biological replicate. Three biological replicates were analyzed. *n* is shown in the panel. Two-tailed Student’s *t*-test: **P < 0.01, ***P < 0.001. Source data

Ptr*VCS2* has one homologue, Ptr*VCS2-h* (Potri. 017G082700) (Extended Data Fig. 2a,b), which is not a VCS gene but is highly expressed in the cambium, xylem and phloem, with a cambium expression level as high as that of Ptr*VCS2* (Extended Data Fig. 2c). Neither Ptr*VCS2* nor Ptr*VCS2-h* had been previously studied. The same phenotypic changes in *OE*-Ptr*VCS2* were also observed in *OE*-Ptr*VCS2-h* (Extended Data Fig. 1c,e–j, Supplementary Fig. 3 and Supplementary Text), suggesting redundant functions for Ptr*VCS2* and Ptr*VCS2-h*; these were also supported by their loss-of-function mutation in *P. trichocarpa*. CRISPR-edited single-knockout ptr*vcs2* and the WT exhibited similar phenotypes (Fig. 2e, Extended Data Fig. 3a–c and Supplementary Text). However, double-knockout ptr*vcs2* ptr*vcs2-h* plants (Fig. 2e and Extended Data Fig. 3d; lines #1 and #2 were analysed) had increased stem diameter (Fig. 2f and Supplementary Fig. 7). The stem internodes examined (5th to 8th) in both double-knockout lines exhibited two to four more cambium cell layers than the WT (Fig. 2g,h, Extended Data Fig. 3e,f and Supplementary Text). When compared with the internodes of the same age (30-day growth period), the cell layer increase persisted in the two tested double mutant lines (Supplementary Fig. 3). The contrasting development in cambium cell layers between the gain- and loss-of-function transgenics suggests a unique function for Ptr*VCS2* in regulating cell proliferation in vascular cambium.

### Ptr*VCS2* represses Ptr*WOX4a* expression in cambium development

**Fig. 3:**
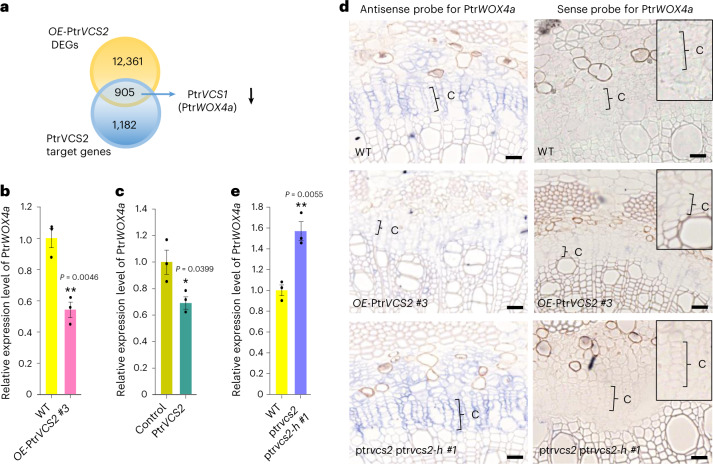
Ptr*VCS2* regulates Ptr*WOX4a* expression in the vascular cambium. **a**, Venn diagram showing the common genes between RNA-seq DEGs (FDR < 0.05) of *OE-*Ptr*VCS2* transgenics and PtrVCS2 target genes (*P* < 1 × 10^−5^). The downward arrow indicates the downregulation of Ptr*VCS1* (Ptr*WOX4a*) by Ptr*VCS2* overexpression. **b**,**e**, Relative expression levels of Ptr*WOX4a* in the cambium of *OE-*Ptr*VCS2* transgenics (**b**) and ptr*vcs2* ptr*vcs2-h* mutants (**e**) were determined by RT–qPCR. The data are shown as mean ± s.e.m.; *n* = 3 biological replicates (**P < 0.01, two-tailed Student’s *t*-test). **c**, Relative expression levels of Ptr*WOX4a* in stem xylem protoplasts overexpressing *GFP* (control) or Ptr*VCS2*. The data are shown as mean ± s.e.m.; *n* = 3 biological replicates (three independent batches of stem xylem protoplast transfections). The asterisk indicates a significant difference between control protoplasts and the samples overexpressing Ptr*VCS2* (*P < 0.05, two-tailed Student’s *t*-test). **d**, In situ hybridization of Ptr*WOX4a* mRNA in the WT, *OE-*Ptr*VCS2* transgenics and ptr*vcs2* ptr*vcs2-h* mutants. Paraffin sections are from the 6th internode of *P. trichocarpa* stems. The black brackets mark vascular cambium cells in one radial cell file. Scale bars, 25 µm. Source data

**Extended Data Fig. 4:**
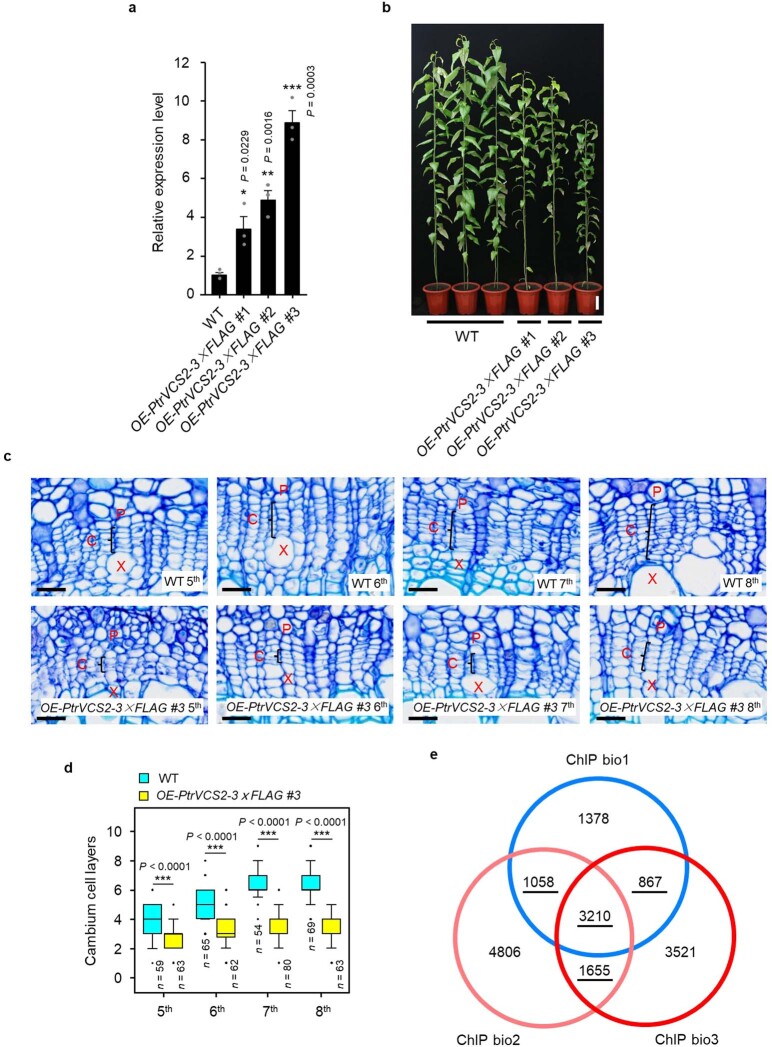
Cambium phenotype analysis of *OE-PtrVCS2-3×FLAG* transgenics and ChIP-seq analysis of *OE-PtrVCS2-3×FLAG* transgenics. **a**, Relative expression levels of *PtrVCS2* in WT and *OE-PtrVCS2-3×FLAG* transgenic plants, as determined by RT-qPCR. The data are shown as mean±s.e.m.; *n* = 3 biological replicates, and asterisks indicate significant differences between each line of the transgenics and WT plants (Two-tailed Student’s *t*-test: *P < 0.05, **P < 0.01, ***P < 0.001). **b**, Phenotypes of WT and *OE-PtrVCS2-3×FLAG* transgenics. Scale bars, 10 cm. **c**, Histochemistry and histological analysis of WT and *OE-PtrVCS2-3×FLAG* #3 transgenics. Cross-sections of the 5th–8th internodes of *P. trichocarpa* stems were stained with toluidine blue O. Black brackets mark the cambium cells in one radial cell file. Scale bars, 25 µm. C, cambium; P, phloem, X, xylem. **d**, Number of cambium cell layers in stem vascular tissues of WT and *OE-PtrVCS2-3×FLAG #3* plants. The number of cambium cell layers of at least ten radial cell files was counted within one cross-section from each biological replicate. Three biological replicates were analyzed. *n* is shown in the panel. Boxes show the median with the upper and lower quantiles, and the whiskers represent the data range excluding outliers. Two-tailed Student’s *t*-test: ***P < 0.001. **e**, Venn diagram showing the common PtrVCS2 binding peaks among three biological replicates (bio) of ChIP-seq analysis. The 6790 reproducible peaks in two or three biological replicates are underlined. Source data

We next performed RNA-sequencing (RNA-seq) analysis on the WT and *OE*-Ptr*VCS2*, which revealed that PtrVCS2 regulates 13,266 genes (false discovery rate (FDR) < 0.05; Supplementary Table 2, Supplementary Text and Methods). We also conducted chromatin immunoprecipitation sequencing (ChIP-seq) on *OE*-Ptr*VCS2* transgenics (Supplementary Text and Extended Data Fig. 4a–d) and identified 6,790 PtrVCS2 binding sites (*P* < 1 × 10^−5^; Extended Data Fig. 4e and Supplementary Table 3a,b) representing 2,087 putative PtrVCS2 target genes with one or more PtrVCS2 binding sites within the 3-kb promoter region (Supplementary Table 3c and Methods). Integrative analysis of ChIP-seq (2,087 targets) and RNA-seq (13,266 differentially expressed genes (DEGs)) suggested that 905 genes are transcriptionally repressed or activated by PtrVCS2 through TF–DNA binding (Fig. 3a).

One telling result of the integrative analysis is that Ptr*VCS2* (the second most abundant *VCS*) could directly *trans*-repress the most abundant *VCS*, Ptr*VCS1* (denoted as Ptr*WOX4a* hereafter) (Fig. 3a). The overexpression of Ptr*VCS2* repressed cambium’s Ptr*WOX4a* expression by nearly one half, compared with the WT (Fig. 3b) and reduced Ptr*WOX4a* RNA signals (Fig. 3d). It could be argued that, despite the reduced RNA signals (Fig. 3d), the PCR with reverse transcription (RT–PCR) based reduction of Ptr*WOX4a* transcript level in *OE-*Ptr*VCS2* cambium could be due to fewer cambium cells in these transgenics than in the WT. We then tested this using a *P*. *trichocarpa* stem xylem protoplast system^42,43^ and demonstrated that the overexpression of Ptr*VCS2* repressed Ptr*WOX4a* expression (Fig. 3c), revealing that the reduced Ptr*WOX4a* expression in *OE-*Ptr*VCS2* was mediated by the PtrVCS2 function rather than fewer cells. When PtrVCS2 functions were eliminated through double mutation of Ptr*VCS2* and Ptr*VCS2-h* (ptr*vcs2* ptr*vcs2-h* in Fig. 2e and Extended Data Fig. 3d), cambium’s Ptr*WOX4a* transcript levels increased by approximately 1.6-fold (Fig. 3e) with a slight increase in Ptr*WOX4a* RNA signals (Fig. 3d).

We next asked (1) whether increased Ptr*WOX4a* transcript levels would result in altered cambium proliferation as observed in ptr*vcs2* ptr*vcs2-h* and (2) whether loss of function in Ptr*WOX4a* would yield cambium systems resembling those in gain of function in Ptr*VCS2* or Ptr*VCS2-h*. To address these questions, we performed transgenesis in Ptr*WOX4*.

### Ptr*WOX4* is required for promoting cambium cell proliferation

**Fig. 4:**
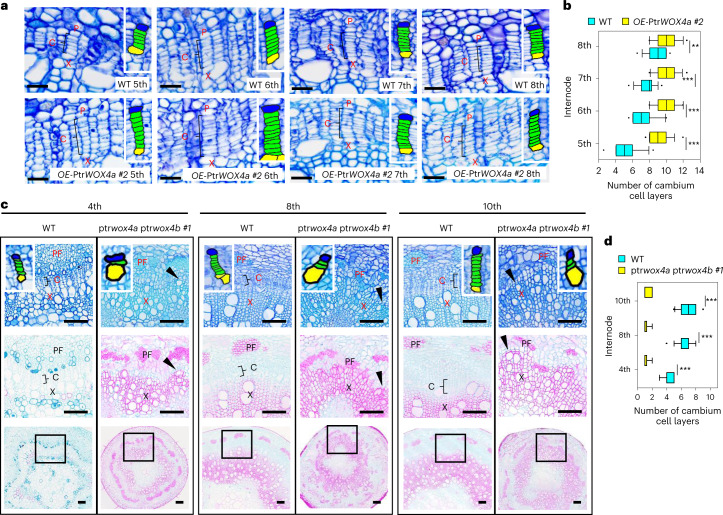
Ptr*WOX4* is crucial for vascular cambium proliferation in *P. trichocarpa*. **a**, Histochemistry and histological analysis of the WT and *OE-*Ptr*WOX4a #2* transgenics. Cross-sections of the 5th–8th internodes of *P. trichocarpa* stems were stained with toluidine blue O. The black brackets mark the cambium cells in one radial cell file. The insets show close-ups of cambium cells (green), adjacent phloem cells (blue) and adjacent xylem cells (yellow). Scale bars, 25 µm. **b**, Number of cambium cell layers in stem vascular tissues of the WT and *OE-*Ptr*WOX4a #2* transgenics. **c**, Histochemistry and histological analysis of the WT and the ptr*wox4a* ptr*wox4b #1* mutants. Cross-sections of the 4th, 8th and 10th internodes of *P. trichocarpa* stems were stained with toluidine blue O, safranine O and fast green. The black brackets for the WT and black arrowheads for the ptr*wox4a* ptr*wox4b* mutants mark the cambium cells in one radial cell file. The insets in the sections stained with toluidine blue O show close-ups of cambium cells (green), adjacent phloem cells (blue) and adjacent xylem cells (yellow). In each column, the image in the middle panel is a magnification of the region marked by a black box from the section stained with safranine O and fast green in the lower panel. Scale bars, 100 µm. PF, phloem fibre. **d**, Number of cambium cell layers in stem vascular tissues of the WT and the ptr*wox4a* ptr*wox4b #1* mutants. In **b** and **d**, the number of cambium cell layers of ten radial cell files was counted within one cross-section from each biological replicate. Three biological replicates were analysed. *n* = 30. The boxes show the median and the upper and lower quantiles, and the whiskers represent the data range excluding outliers. Two-tailed Student’s *t*-test: **P < 0.01; ***P < 0.001. The *P* values versus the WT control for *OE*-Ptr*WOX4a #2* transgenics in **b** are as follows: 5th, <0.0001; 6th, <0.0001; 7th, <0.0001; 8th, 0.0031. The *P* values versus the WT control for ptr*wox4a* ptr*wox4b #1* mutants in **d** are as follows: 4th, <0.0001; 8th, <0.0001; 10th, <0.0001. Source data

**Extended Data Fig. 5:**
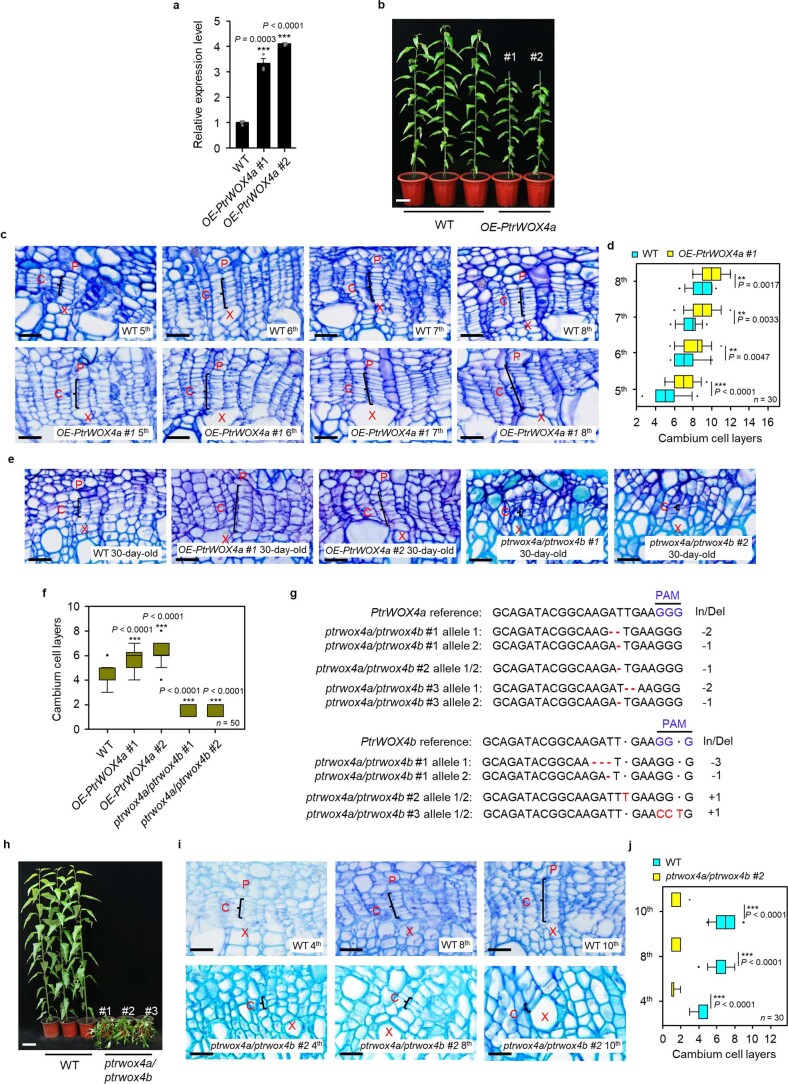
Identification of *OE-PtrWOX4a* transgenics and double-knockout *ptrwox4a/ptrwox4b* mutants and analysis of their cambium phenotypes. **a**, Relative expression levels of the *PtrWOX4a* transgene in the cambium of independent *OE-PtrWOX4a* transgenic lines, as determined by RT-qPCR. The data are shown as mean±s.e.m.; *n* = 3 technical replicates, two-tailed Student’s *t*-test: ***P < 0.001. **b**,**h**, Phenotypes of WT, *OE*-*PtrWOX4a* transgenics (**b**) and *ptrwox4a/ptrwox4b* mutants (**h**). Scale bars, 10 cm. **c**,**i**, Histochemistry and histological analysis of the WT, *OE-PtrWOX4a #1* transgenics (**c**) and *ptrwox4a/ptrwox4b #2* mutants (**i**). **d**,**j**, Number of cambium cell layers in stem vascular tissues of WT, *OE-PtrWOX4a #1* transgenics (**d**) and *ptrwox4a/ptrwox4b #2* mutants (**j**). **e**, Histochemistry and histological analysis of the same age internodes (30-day growth) from WT, *OE-PtrWOX4a* transgenics and *ptrwox4a/ptrwox4b* mutants. **f**, Number of cambium cell layers in stem vascular tissues of the same age internodes (30-day growth) from WT, *OE-PtrWOX4a* transgenics and *ptrwox4a/ptrwox4b* mutants. **g**, Mutations at the sgRNA target sites for *PtrWOX4a* and *PtrWOX4b* in three independent *ptrwox4a/ptrwox4b* mutant lines. Deleted nucleotides are depicted as red dashes, and substitutions or inserted nucleotides are represented in red font. Blue font indicates the protospacer-adjacent motif (PAM), and the nucleotide lengths of insertions and/or deletions (In/Del) are presented on the right. In (**c**), (**e**) and (**i**), cross-sections of *P. trichocarpa* stem internodes were stained with toluidine blue O. Black brackets mark the cambium cells in one radial cell file. Scale bars, 25 µm. C, cambium; P, phloem; X, xylem. In (**d**), (**f**) and (**j**), cambium cell layer numbers of at least ten radial cell files were counted within one cross-section from each biological replicate. Three biological replicates were carried out. *n* is shown in the panel. Boxes show the median with the upper and lower quantiles, and the whiskers represent the data range excluding outliers. Two-tailed Student’s *t*-test: **P < 0.01, ***P < 0.001. Source data

Like ptr*vcs2* ptr*vcs2-h* (Fig. 2g,h, Extended Data Fig. 3e,f and Supplementary Fig. 3), the two *OE*-Ptr*WOX4a* transgenics generated (lines #1 and #2; Extended Data Fig. 5a,b) had four to six more cell layers in their stem vascular cambium than the WT (5th–8th internodes of all plants; Fig. 4a,b and Extended Data Fig. 5c,d). When compared with the internodes of the same age (30-day growth), the cell layer addition persisted in the two tested *OE*-Ptr*WOX4a* lines (Extended Data Fig. 5e,f). The CRISPR double mutations in Ptr*WOX4a* and Ptr*WOX4b* (Extended Data Fig. 5g,h) severely disrupted the normal vascular cambium development, leaving the cambium zone with only one to two cell layers—that is, an elimination of six to eight cell layers (4th–10th internodes of all plants; line #1 in Fig. 4c,d and line #2 in Extended Data Fig. 5i,j). The cell layer elimination persisted in the same-aged (30-day growth) stem internodes in the two tested double mutant lines (Extended Data Fig. 5e,f). These results are consistent with the reduction of four to six cambium cell layers in *OE-*Ptr*VCS2* transgenics where Ptr*WOX4a* was partially repressed (Fig. 3b). These gain/loss-of-function and phenotype results may suggest regulatory associations between Ptr*VCS2* and Ptr*WOX4a*. We next investigated this possible regulatory association.

The presence of a ZF but the lack of an HD in PtrVCS2 (Supplementary Text) and integrated RNA-seq/ChIP-seq analysis suggest that PtrVCS2 may *trans*-activate or *trans*-repress its target genes (Fig. 3a, Supplementary Fig. 9 and Supplementary Table 3) by interacting with other HD-bearing TFs that can directly bind to such targets, such as Ptr*WOX4*. We then searched for PtrVCS2’s possible direct interactive partners through yeast two-hybrid (Y2H) screening of 59 PtrVCS TFs (Supplementary Table 4a) that we had cloned (Methods).

**Fig. 5:**
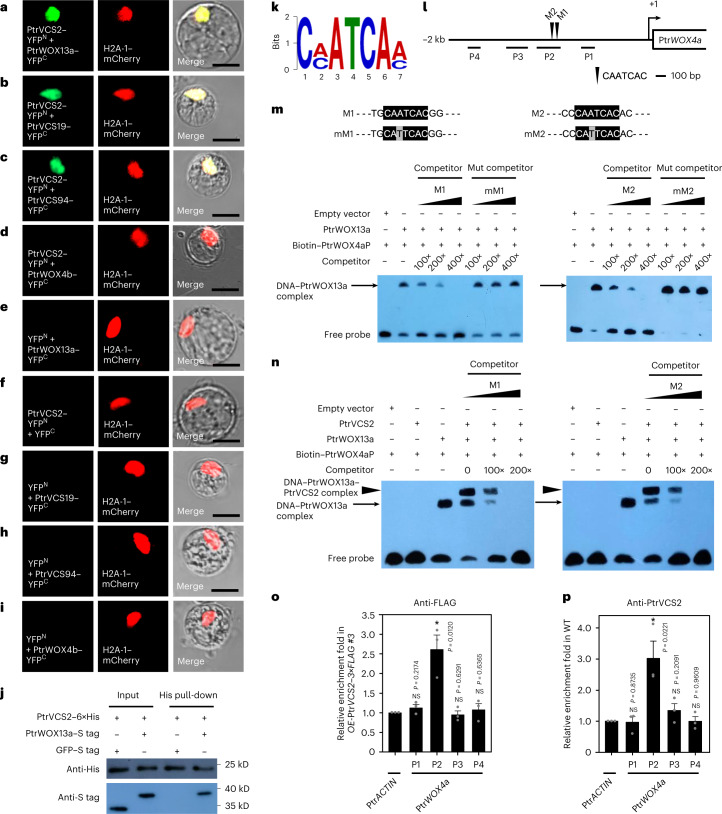
PtrVCS2 is recruited to the Ptr*WOX4a* promoter through dimerization with PtrWOX13a. **a**–**i**, BiFC assays in *P. trichocarpa* stem-differentiating xylem (SDX) protoplasts showing that PtrVCS2 interacts with PtrWOX13a (**a**), PtrVCS19 (**b**) and PtrVCS94 (**c**) but not PtrWOX4b (**d**) in vivo. Each BiFC pair of constructs was co-transfected with the *H2A-1–mCherry* nuclear marker construct. Co-transfection of each construct of interest with empty plasmid served as controls (**e**–**i**). Scale bars, 10 μm. **j**, Interaction of PtrWOX13a–PtrVCS2 dimer determined by pull-down assays. **k**, MEME-ChIP analysis identifies C(A/C)ATCA(A/C) as a statistically defined motif (*e*-value, 5.2 × 10^−10^). A total of 6,790 PtrVCS2 binding peaks identified from three biological replicates of ChIP experiments were used for MEME-ChIP analysis. **l**, Schematic diagram of the WOX13 binding motif in the Ptr*WOX4a* promotor. **m**, EMSA showing that PtrWOX13a binds to the CAATCAC motif in the Ptr*WOX4a* promoter. **n**, PtrVCS2 alone fails to bind to the CAATCAC motif in the Ptr*WOX4a* promoter, and PtrWOX13a is required for the association of PtrVCS2 with the Ptr*WOX4a* promoter. In **m** and **n**, the nucleotide sequences of the WT M1 and M2 and the mutated M1 (mM1) and M2 (mM2) are shown. The core sequences are shaded in black, and the mutated nucleotide is shaded in grey. Unlabelled Ptr*WOX4a* promoter fragments were used as competitors. Empty vector (pET101-His) was used as a negative control. **o**,**p**, ChIP–qPCR assays showing that PtrVCS2 associates with the Ptr*WOX4a* promoter. Transgenic plants overexpressing 3×FLAG (control) or PtrVCS2–3×FLAG were used for the ChIP assays with anti-FLAG antibody in **o**. WT plants were used for the ChIP analysis with anti-PtrVCS2 antibody in **p**, and anti-IgG antibody was used as a control. Enrichment of DNA was calculated as the ratio between PtrVCS2–3×FLAG and 3×FLAG or between anti-PtrVCS2 antibody and anti-IgG antibody, normalized to that of the Ptr*ACTIN* gene. The data are shown as mean ± s.e.m.; *n* = 3 biological replicates; two-tailed Student’s *t*-test; *P < 0.05; NS, not significant. The experiments in **a**–**j**,**m**,**n** were repeated independently three times, with consistent results. Source data

**Extended Data Fig. 6:**
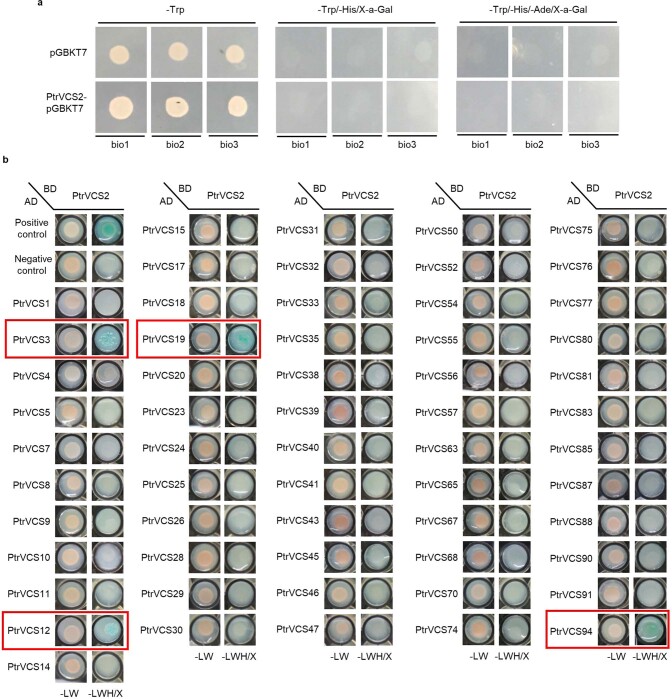
Y2H screening of PtrVCS2 for PtrVCS TFs. **a**, The full-length coding sequence of *PtrVCS2* in the pGBKT7 BD vector (PtrVCS2-pGBKT7) was used for transcriptional activation assay. There was no transcriptional activation activity for the full-length PtrVCS2. **b**, PtrVCS2 was fused to the DNA binding domain (BD) of GAL4 in the pGBKT7 vector, and PtrVCS TFs were fused to the GAL4 activation domain (AD) in the pGADT7 vector. Each bait and prey pair were co-transformed in yeast cells and selected on –LW: SD/–Leu/–Trp and –LWH/X: SD/–Leu/–Trp/–His/X-α-Gal medium. Red boxes show interactions between PtrVCS2 and its interacting proteins. BD-53/AD-Lam was used as the negative control and BD-53/AD-T as the positive control. The experiments were repeated independently three times with consistent results.

**Extended Data Fig. 7:**
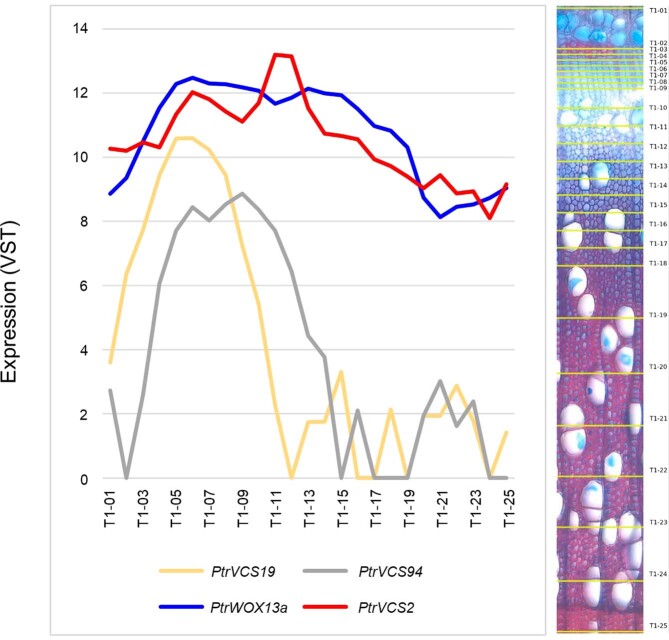
Expression profiles of *PtrVCS2* and its interaction partners. Expression profiles of *PtrVCS2* and PtrVCS2-interacting *PtrVCS* TF genes across secondary stem tissues based on the RNA-seq datasets of trees^45^. The horizontal axis corresponds to the transverse cross-section shown to the right, which is modified from AspWood (http://aspwood.popgenie.org).

We identified four PtrVCS TF proteins—PtrVCS3 (identical to PtrWOX4b^30^), PtrVCS12 (identical to PtrWOX13a^44^), PtrVCS19 and PtrVCS94—that could interact with PtrVCS2 in yeast (Extended Data Fig. 6 and Supplementary Table 4a). Bimolecular fluorescence complementation (BiFC) assays validated that PtrVCS2 could dimerize with three of these TFs in planta: PtrWOX13a, PtrVCS19 and PtrVCS94 (Fig. 5a–i). Among these three, Ptr*WOX13a* exhibited expression patterns very similar to those of Ptr*VCS2* throughout cambium and xylem development^45^ (Extended Data Fig. 7), suggesting that PtrWOX13a may be a more committed and synchronized functional PtrVCS2 partner than PtrVCS19 or PtrVCS94. The interaction between PtrWOX13a and PtrVCS2 (denoted as W13–V) was further confirmed by in vitro pull-down assay (Fig. 5j). PtrWOX13a may therefore be the HD-bearing TF bridging the association between PtrVCS2 and PtrWOX4a, as we suggested above.

### PtrVCS2 is recruited to Ptr*WOX4a* by interacting with PtrWOX13a

We analysed the DNA sequences flanking all peak summits identified from ChIP-seq for PtrVCS2 and identified C(A/C)ATCA(A/C) as one of the top-ranked motifs in the Ptr*WOX4a* promoter for TF binding (*e*-value, 5.2 × 10^−10^; Fig. 5k and Methods). This motif is highly similar to the *cis*-regulatory sequences that WOX13 would bind to for *trans*-regulation in plants^46^. There are two such CAATCAC binding sites (M1 and M2 in the P2 fragment; Fig. 5l) in the Ptr*WOX4a* promoter within the 2-kb sequences upstream of the transcription start site.

**Extended Data Fig. 8:**
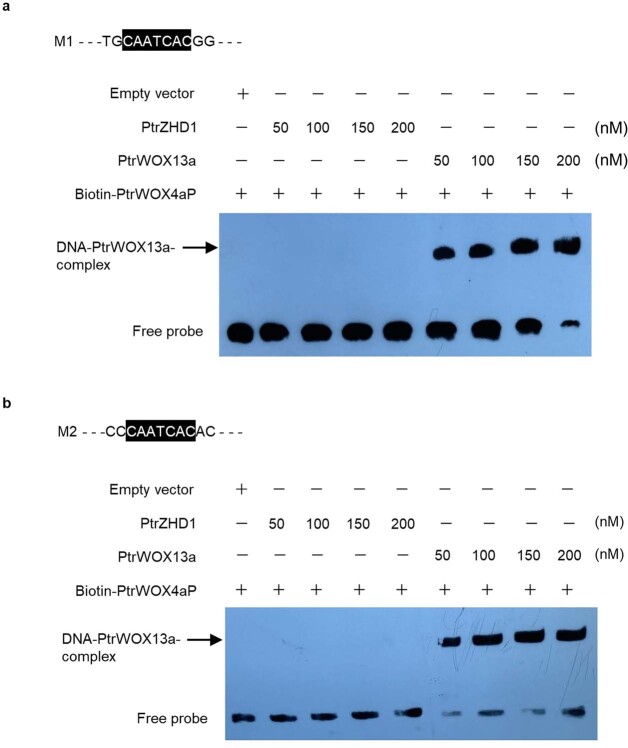
EMSAs show that the binding of PtrWOX13a to the CAATCAC motif in the promoter of *PtrWOX4a* is specific. The empty vector (PET101-His) was used as a negative control. The homeodomain transcription factor PtrZFD1 (Potri.002G035200) did not bind to the CAATCAC motif at a gradient of protein concentrations, which further confirmed the binding specificity of PtrWOX13a. The experiments in **a** and **b** were repeated independently three times with similar results. Source data

We next performed electrophoretic mobility shift assays (EMSAs) and demonstrated that PtrWOX13a binds directly to the Ptr*WOX4a* promoter’s M1 and M2 sites (Fig. 5m). The binding requiring the CAATCAC motif was demonstrated by competition assays with single-nucleotide-mutated M1 and M2 competitors (Fig. 5m). The EMSA results with a negative control (an HD-bearing protein, PtrZHD1 (Potri.002G035200)) at high concentrations supported PtrWOX13a’s binding specificity to Ptr*WOX4a* (Extended Data Fig. 8). This TF–DNA binding suggested a gene *trans*-regulation function, as the overexpression of Ptr*WOX13a* in *P*. *trichocarpa* stem xylem protoplasts^42,43^ doubled the Ptr*WOX4a* transcript level (Supplementary Fig. 10a). Glucocorticoid-receptor-based inducible gene expression assays^47^ confirmed direct regulation of the Ptr*WOX4a* transcription by PtrWOX13a (Supplementary Fig. 10b–d). Further EMSA experiments demonstrated that PtrVCS2 could not bind to the Ptr*WOX4a* promoter’s M1 and M2 sites, but PtrWOX13a–PtrVCS2 protein dimers could (Fig. 5n). EMSAs thus supported PtrWOX13a’s binding specificity to Ptr*WOX4a* as individual proteins or PtrWOX13a–PtrVCS2 protein dimers, suggesting a *trans*-regulatory association between PtrVCS2 and Ptr*WOX4a* through indirect TF–target gene binding.

We then performed ChIP with quantitative PCR (qPCR) on stem vascular cambium of transgenics overexpressing Ptr*VCS2* tagged with *FLAG* (*OE-*Ptr*VCS2–3*×*FLAG*; Extended Data Fig. 4a–d) using anti-FLAG antibodies and detected a ~2.5-fold enrichment of the M1 and M2 motif-containing P2 promoter fragment of Ptr*WOX4a* (Fig. 5l,o). Using stem vascular cambium of WT *P. trichocarpa* plants for ChIP–qPCR with anti-PtrVCS2 antibodies, we obtained similar results on the enrichment of the Ptr*WOX4a* fragments (Fig. 5l,p). Therefore, in vitro and in vivo (both transgenic and WT plants) evidence supports an association between PtrVCS2 and Ptr*WOX4a* through indirect protein–Ptr*WOX4a* binding specifically to the P2 promoter fragment via PtrWOX13a–PtrVCS2 protein dimers.

Overall, these results suggest that PtrVCS2 is recruited to Ptr*WOX4a* (Fig. 5o,p and Supplementary Table 3) through its interaction with PtrWOX13a (Fig. 5a,e,f,j), which directly binds to the M1 and M2 motifs of the Ptr*WOX4a* promoter (Fig. 5l–n). Our results suggest a Ptr*VCS2*–Ptr*WOX13a*–Ptr*WOX4a* regulatory system.

### PtrVCS2 regulates Ptr*WOX4a* through PtrWOX13a and the histone acetyltransferase complex

**Fig. 6:**
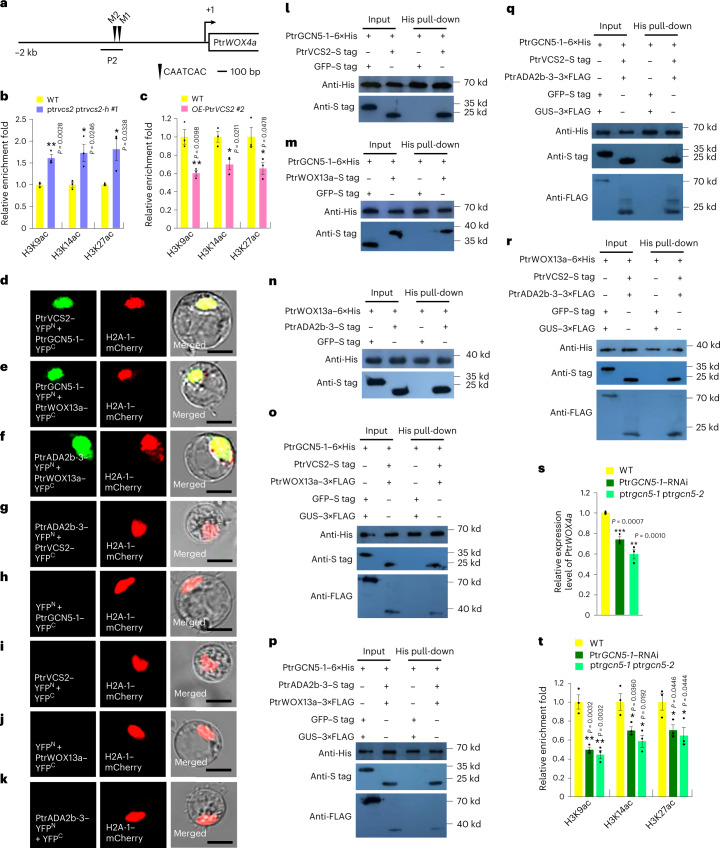
PtrVCS2 regulates the histone acetylation levels at the Ptr*WOX4a* promoter via the PtrWOX13a–PtrVCS2–PtrGCN5-1–PtrADA2b-3 tetrameric protein complex. **a**, Schematic diagram of the CAATCAC motif (the WOX13-binding motif) in the Ptr*WOX4a* promoter. **b**,**c**, Relative enrichment fold of H3K9ac, H3K14ac and H3K27ac at the Ptr*WOX4a* promoter in the ptr*vcs2* ptr*vcs2-h #1* mutants (**b**) and *OE*-Ptr*VCS2 #2* transgenics (**c**). **d**–**k**, BiFC assays in *P. trichocarpa* SDX protoplasts showing that PtrGCN5-1 interacts with PtrVCS2 (**d**) and PtrWOX13a (**e**) and that PtrADA2b-3 interacts with PtrWOX13a (**f**) but not with PtrVCS2 (**g**). Each BiFC pair of constructs was co-transfected with the *H2A-1–mCherry* nuclear marker construct. Co-transfection of each construct of interest with empty plasmid served as a control (**h**–**k**). Scale bars, 10 μm. **l**–**n**, Interactions of PtrVCS2–PtrGCN5-1 (**l**), PtrWOX13a–PtrGCN5-1 (**m**) and PtrADA2b-3–PtrWOX13a (**n**) dimers, as determined by pull-down assays. **o**–**r**, Interactions of PtrWOX13a–PtrVCS2–PtrGCN5-1 (**o**), PtrWOX13a–PtrGCN5-1–PtrADA2b-3 (**p**), PtrVCS2–PtrGCN5-1–PtrADA2b-3 (**q**) and PtrADA2b-3–PtrWOX13a–PtrVCS2 (**r**) trimers, as determined by pull-down assays. **s**, Relative expression levels of Ptr*WOX4a* in Ptr*GCN5-1*–RNAi transgenics and the ptr*gcn5-1* ptr*gcn5-2* mutants, as determined by RT–qPCR. The data are shown as mean ± s.e.m.; *n* = 3 biological replicates; two-tailed Student’s *t*-test; **P < 0.01; ***P < 0.001. **t**, Relative enrichment fold of H3K9ac, H3K14ac and H3K27ac at the Ptr*WOX4a* promoter in Ptr*GCN5-1*–RNAi transgenics and the ptr*gcn5-1* ptr*gcn5-2* mutants. In **b**, **c** and **t**, ChIP assays were performed using antibodies against H3K9ac, H3K14ac and H3K27ac, and the precipitated DNA was quantified by qPCR. Enrichment values represent the relative fold change compared with WT plants. The data are shown as mean ± s.e.m.; *n* = 3 biological replicates, and the asterisks indicate significant differences between each transgenic line and WT plants (two-tailed Student’s *t*-test; *P < 0.05; **P < 0.01). The experiments in **d**–**r** were repeated independently three times, with consistent results. Source data

**Extended Data Fig. 9:**
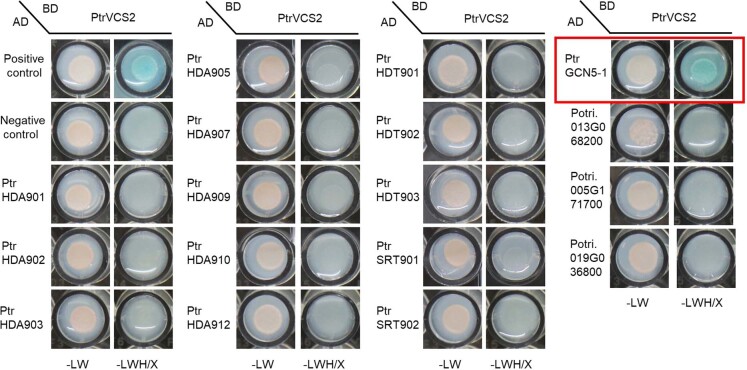
Y2H assays between PtrVCS2 and HAT or HDAC proteins. PtrVCS2 was fused to the DNA binding domain (BD) of GAL4 in the pGBKT7 vector and HATs or HDACs were fused to the GAL4 activation domain (AD) in the pGADT7 vector. Each bait and prey construct were co-transformed in yeast cells and selected on –LW: SD/–Leu/–Trp and –LWH/X: SD/–Leu/–Trp/–His/X-α-Gal medium. The red boxes showed the interaction between PtrVCS2 and PtrGCN5-1. BD-53/AD-Lam was used as a negative control and BD-53/AD-T was used as a positive control. The experiments were repeated independently three times with consistent results.

The presence of a Ptr*VCS2*–Ptr*WOX13a*–Ptr*WOX4a* regulatory system suggests that PtrVCS2 may repress Ptr*WOX4a* gene expression through this pathway to regulate cambium development (Figs. 3 and 5). Because levels of epi-markers may influence gene expression, we compared levels of acetylated lysine residues 9, 14 and 27 of histone H3 (H3K9ac, H3K14ac and H3K27ac) at the Ptr*WOX4a* promoter between the WT and ptr*vcs2* ptr*vcs2-h* double mutants, and between the WT and *OE-*Ptr*VCS2* transgenics. ChIP–qPCR analysis revealed that the levels of the three epi-markers at the P2 Ptr*WOX4a* promoter fragment were substantially increased in the double mutants (Fig. 6a,b) and decreased in the *OE-*Ptr*VCS2* transgenics (Fig. 6a,c), suggesting that PtrVCS2 may regulate the expression of Ptr*WOX4a* through its effects on histone acetylation. We then cloned 13 *P*. *trichocarpa* histone deacetylase genes and 4 histone acetyltransferase (HAT) genes (Supplementary Table 4b) for Y2H screening of interactions between PtrVCS2 and these acetylation effectors. We found no interactions with histone deacetylase, but one HAT, PtrGCN5-1 (ref. 48) (*P. trichocarpa* GENERAL CONTROL NON-DEREPRESSIBLE5-1), interacted with PtrVCS2 (Extended Data Fig. 9). BiFC (Fig. 6d,h,i) and in vitro pull-down (Fig. 6l) experiments confirmed the PtrVCS2–PtrGCN5-1 (denoted as V–G) interaction and the interaction specificity in the nucleus in vivo. We also found that PtrWOX13a interacted with PtrGCN5-1 (denoted as W13–G; Fig. 6e,h,j,m).

GCN5, the subunit of a HAT, dimerizes with ALTERATION/DEFICIENCY IN ACTIVATION2 (ADA2) to confer HAT catalytic activity^49–51^. We have previously confirmed a PtrGCN5-1–PtrADA2b-3 complex (denoted as G–A) for HAT functions in *P*. *trichocarpa*^48^. Here, we observed the formation of nuclear dimers of PtrADA2b-3–PtrWOX13a using BiFC (denoted as A–W13; Fig. 6f,j,k), but we detected no interaction between PtrVCS2 and PtrADA2b-3 (Fig. 6g,i,k). The interaction between PtrADA2b-3 and PtrWOX13a was further confirmed by in vitro pull-down (Fig. 6n). We identified the PtrWOX13a–PtrVCS2 dimer above (W13–V; Fig. 5a,e,f,j). These five sets of dimers (W13–V, V–G, W13–G, G–A and A–W13) each having components that can couple with another two of the four proteins may suggest interactions for a tetrameric protein complex, W13–V–G–A. Further in vitro pull-down experiments demonstrated the formation of all the possible trimers, W13–V–G (Fig. 6o), W13–G–A (Fig. 6p), V–G–A (Fig. 6q) and A–W13–V (Fig. 6r), associated with the tetramer. All protein interaction results indicate that in this W13–V–G–A tetramer, V interacts with W13–G–A through W13 (Fig. 5a,e,f,j) and G (Fig. 6d,h,i,l) but not through A (as there is no interaction between V and A; Fig. 6g,i,k).

### PtrVCS2 acts as a suppressor of the HAT complex functions

**Extended Data Fig. 10:**
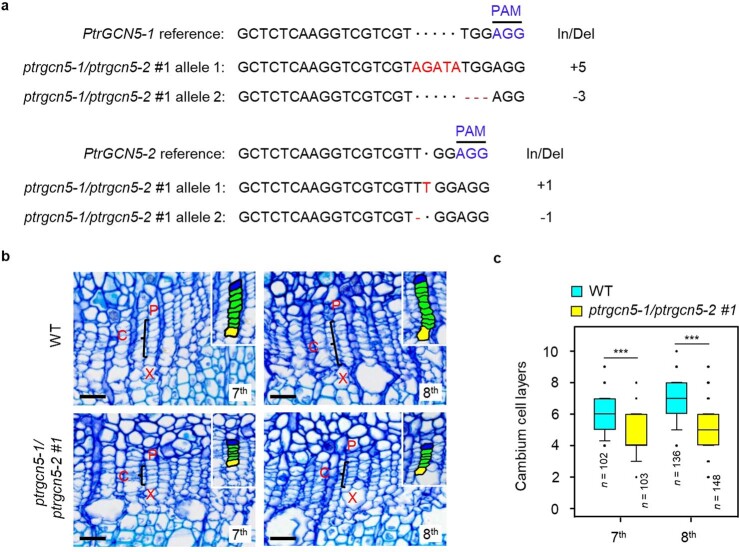
Identification of *ptrgcn5-1/ptrgcn5-2* double mutants and analysis of the cambium phenotypes. **a**, Mutations at the sgRNA target sites for *PtrGCN5-1* and *PtrCN5-2* in the *ptrgcn5-1/ptrgcn5-2* mutant. Deleted nucleotides are depicted as red dashes, and substitutions or inserted nucleotides are represented in red font. Blue font indicates the protospacer-adjacent motif (PAM), and the nucleotide lengths of insertions and/or deletions (In/Del) are shown on the right. **b**, Histochemistry and histological analysis of WT and *ptrgcn5-1/ptrgcn5-2 #1* mutants. Cross-sections of the 7th and 8th internodes of *P. trichocarpa* stems were stained with toluidine blue O. Black brackets mark the cambium cells in one radial cell file. Insets show closeups of the cambium cells (green), adjacent phloem cells (blue) and adjacent xylem cells (yellow). Scale bars, 25 µm. **c**, Number of cambium cell layers in stem vascular tissues of WT and *ptrgcn5-1/ptrgcn5-2 #1* mutant. Cambium cell layer numbers of at least ten radial cell files were counted within one cross-section from each biological replicate. Three biological replicates were carried out. *n* is shown in the panel. Boxes show the median with the upper and lower quantiles, and the whiskers represent the data range excluding outliers. Asterisks indicate significant difference between the mutant and WT plants, ***P < 0.001 (Two-tailed Student’s *t*-test). *P* values versus WT control for *ptrgcn5-1/ptrgcn5-2* mutants are as follows: 7th, <0.0001, 8th, <0.0001. Source data

The binding of the PtrWOX13a–PtrVCS2–PtrGCN5-1–PtrADA2b-3 tetramer to the Ptr*WOX4a* promoter suggests a histone-acetylation-mediated *trans*-regulation of Ptr*WOX4a*. Consistently, in the stem vascular cambium of the ptr*gcn5-1* ptr*gcn5-2* double mutant (Extended Data Fig. 10a) and Ptr*GCN5-1*–RNAi transgenics^48^, we found reduced expression of Ptr*WOX4a* (Fig. 6s) accompanied by drastically decreased histone acetylation (H3K9ac, H3K14ac and H3K27ac) levels at the Ptr*WOX4a* promoter (Fig. 6t) and fewer cambium cell layers (Extended Data Fig. 10b,c). Thus, while Ptr*VCS2* represses Ptr*WOX4a* transcription, PtrGCN5-1 may activate Ptr*WOX4a* through elevated H3K9ac, H3K14ac and H3K27ac markers, revealing regulatory interplays for the PtrWOX13a–PtrVCS2–PtrGCN5-1–PtrADA2b-3 system in controlling Ptr*WOX4a* expression.

**Fig. 7:**
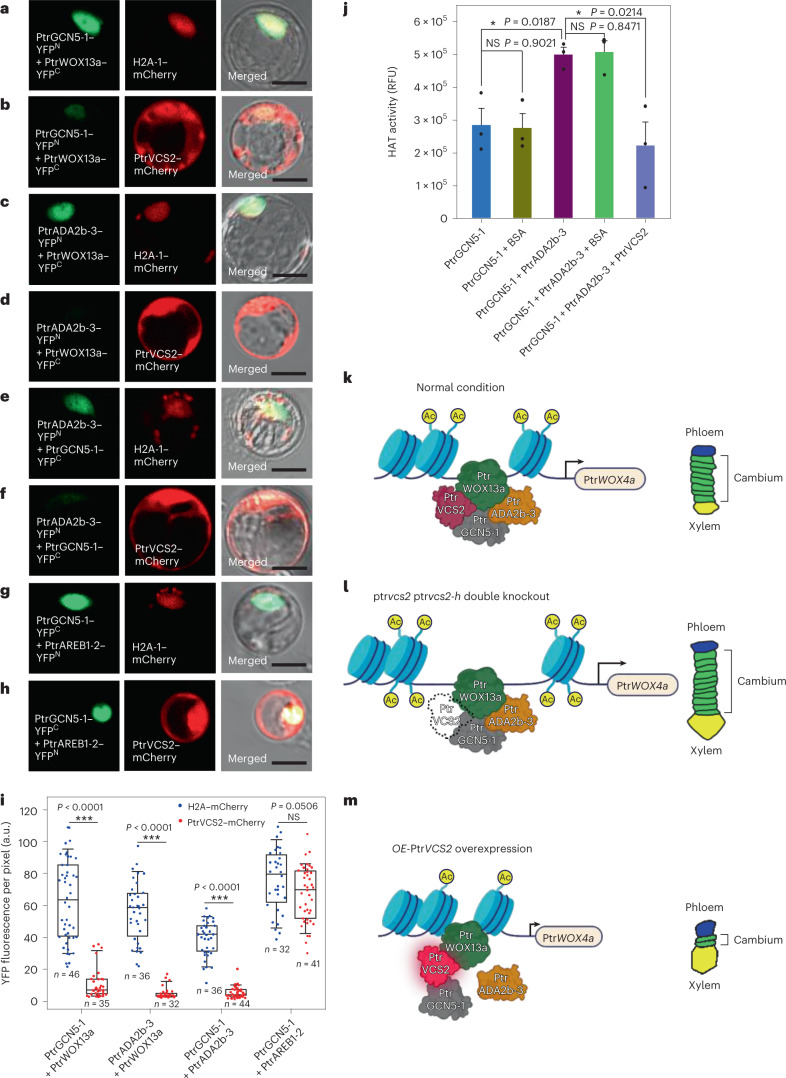
PtrVCS2 reduces the HAT activity of the PtrWOX13a–PtrGCN5-1–PtrADA2b-3 ternary complex by attenuating the three pairwise interactions. **a**–**f**, BiFC assays in *P. trichocarpa* SDX protoplasts showing that PtrVCS2 attenuates the YFP fluorescence signal resulting from the interactions between PtrWOX13a and PtrGCN5-1 (**b**), PtrWOX13a and PtrADA2b-3 (**d**), and PtrGCN5-1 and PtrADA2b-3 (**f**) compared with the control H2A-1–mCherry (**a**,**c**,**e**). **g**,**h**, BiFC assays showing that PtrVCS2 has no effect on the interaction between PtrGCN5-1 and PtrAREB1-2 (ref. 48). All BiFC assays were performed with the same conditions. Scale bars, 10 μm. The experiments in **a**–**h** were repeated independently three times, with similar results. **i**, YFP fluorescence intensity of the interactions between PtrWOX13a and PtrGCN5-1, PtrWOX13a and PtrADA2b-3, PtrGCN5-1 and PtrADA2b-3, and PtrGCN5-1 and PtrAREB1-2 with co-expression of PtrVCS2–mCherry or the control H2A-1–mCherry. a.u., artificial units. Each transformation was performed with three biological replicates. The boxes show the median and the upper and lower quantiles, and the whiskers represent the data range excluding outliers. Two-tailed Student’s *t*-test; ***P < 0.001; NS, not significant. **j**, HAT activity assay. Enzymatic activity was shown as relative fluorescence units (RFU). BSA, bovine serum albumin. The data are shown as mean ± s.e.m.; *n* = 3 biological replicates. Two-tailed Student’s *t*-test; *P < 0.05; NS, not significant. **k**–**m**, Model of the tetramer–Ptr*WOX4a* regulatory pathway and its effects on cambium cell proliferation. Under normal conditions (**k**), Ptr*VCS2* is at normal expression levels, and the tetrameric protein complex allows histone acetylation in Ptr*WOX4a* at normal levels, thereby conferring normal expression levels of Ptr*WOX4a* for the maintenance of normal vascular cambium development. When the expression of Ptr*VCS2* becomes inadequate (**l**), PtrGCN5-1–PtrADA2b-3 HAT activity is activated to hyperacetylate Ptr*WOX4a* and upregulate Ptr*WOX4a* transcription, resulting in an extended proliferation phase with more layers of cambium cells. When the expression of Ptr*VCS2* is elevated (**m**), the interaction of PtrADA2b-3–PtrGCN5-1 is disrupted, thereby reducing the histone acetylation functions of the complex, resulting in suppressed Ptr*WOX4a* expression and thus fewer cambium cell layers. Ac, acetylation; developing phloem cells are shown in blue, developing cambium cells are shown in green and differentiating xylem cells are shown in yellow. Source data

Next, we used BiFC (Fig. 7a–h) to explore whether PtrVCS2 might affect the stability of the PtrWOX13a–PtrGCN5-1–PtrADA2b-3 protein complex. To this end, we fused PtrVCS2 to mCherry and co-transfected the fusion with each of the three BiFC interaction pairs—PtrGCN5-1–YFP^N^ and PtrWOX13a–YFP^C^, PtrADA2b-3–YFP^N^ and PtrWOX13a–YFP^C^, and PtrADA2b-3–YFP^N^ and PtrGCN5-1–YFP^C^—into *P. trichocarpa* xylem protoplasts. All three pairwise interactions were attenuated by PtrVCS2 (Fig. 7a versus 7b, Fig. 7c versus 7d and Fig. 7e versus 7f), as exemplified by the nearly diminished YFP fluorescent intensity (Fig. 7i and Methods). As a negative control, our previously validated interaction between PtrGCN5-1 and PtrAREB1-2 (which is not a paired member of the tetramer^48^) was not affected in the presence of PtrVCS2 (Fig. 7g–i), confirming the specificity of PtrVCS2 in voiding interactions among PtrWOX13a, PtrGCN5-1 and PtrADA2b-3. Without ADA2, GCN5 has inadequate HAT catalytic activity; therefore, the PtrVCS2-mediated disruption of the PtrADA2b-3–PtrGCN5-1 interaction would reduce the ternary complex’s histone acetylation functions.

To test whether PtrVCS2 acts as a suppressor of the ternary’s HAT functions for repressing Ptr*WOX4a* expression, we purified PtrGCN5-1, PtrADA2b-3 and PtrVCS2 recombinant proteins (Methods) for HAT activity assays. We found that PtrGCN5-1 alone or with bovine serum albumin showed only weak HAT activity (Fig. 7j). Strong HAT activities were detected when PtrGCN5-1 and PtrADA2b-3 were present together (Fig. 7j), and such activities were not affected by the addition of bovine serum albumin (Fig. 7j) but were sharply reduced in the presence of PtrVCS2 (Fig. 7j). Overall, our results support a regulatory system involving a tetrameric protein complex that may leverage the levels of histone acetylation of Ptr*WOX4a* for *trans*-regulating the normal development of the vascular cambium for wood formation in *P. trichocarpa*.

## Discussion

We used a cell-type-specific approach to identify 95 stem VCS TFs in *P. trichocarpa*. In *Arabidopsis*, a TF-based regulatory network has been established for vascular cambium development in roots^25^. In this network, At*WOX4* is a major node for the regulation of vascular cambium development^25^. This is an organ- and species-specific network, because many stem-cambium-specific TFs (such as At*WOX14*, *ETHYLENE RESPONSE FACTOR018* (*AtERF018*), *AtERF109*, *AUXIN RESPONSE FACTOR5* (*AtARF5*) and *AtARF7* (refs. 24,52–54)) are not involved in this network. Only four members of this network (Supplementary Table 5) have homologues in the 95 VCS TFs in *P. trichocarpa* stems.

Of the 95 VCS TFs, Ptr*WOX4a* and Ptr*VCS2* are the two most abundant ones. Our work suggests a unique regulatory system coupling these two TFs for the maintenance of normal vascular cambium development in wood formation (Fig. 7k–m). The system consists of a tetrameric protein complex, PtrWOX13a–PtrVCS2–PtrGCN5-1–PtrADA2b-3, which binds directly to Ptr*WOX4a* through PtrWOX13a (Figs. 5 and 6). We analysed the CAATCAC motif in *WOX4* gene promoters in 13 plant species and found that the motif is conserved in 11 (mostly woody plants) of these 13 species (Supplementary Fig. 11). *Arabidopsis* At*WOX4* and rice Os*WOX4* promoters do not have this motif. These results suggest that the CAATCAC motif may form a transacting platform for regulating *WOX4* expression for processes that are more conserved for wood formation. It is possible that this *trans*-regulation may also be mediated by phytohormones such as cytokinin^55^, auxin and gibberellin^56^ that play crucial roles in cambium development in *Populus* species. The hormone mediation may be related to *VCS2* genes, which have been suggested to integrate signals from multiple phytohormones^41^. Further exploration of the connections between the VCS2-mediated *WOX4* *trans*-regulation and hormone signalling should yield new insights into the regulation of cambium development. In this study, we revealed that a tetramer–Ptr*WOX4a* pathway forms a unique epigenetic modification machinery enabling PtrVCS2 to control the dynamics of histone acetylation at Ptr*WOX4a* and thus the dynamics of Ptr*WOX4a* transcription. Epigenetic modifications are key factors influencing many attributes associated with growth and adaptation^48,57,58^. Our work provides insights into how such factors affect vascular cambium development for wood formation.

We suggest that under normal growth conditions, the tetrameric protein–Ptr*WOX4a* network (Fig. 7k) maintains a typical cambium cell proliferation system producing roughly eight cell layers^7,43,59^, defined by the network members’ expression and chromatin histone acetylation levels. When Ptr*VCS2* expression becomes inadequate, PtrGCN5-1–PtrADA2b HAT activities are activated (Fig. 7j) for hyperacetylating Ptr*WOX4a* to upregulate Ptr*WOX4a* transcription, resulting in an extended proliferation phase with more layers of enlarging cambium cells (Fig. 7l). When Ptr*VCS2* expression is elevated, the elevation imparts Ptr*WOX4a* hypoacetylation for suppressed Ptr*WOX4a* function, resulting in reduced cambium cell proliferation and thus fewer cell layers (Fig. 7m). The proliferation of cambium cells affects the progression of the differentiation and maturation of these cells into fibre and vessel elements for the formation of the secondary xylem (wood)^1,2^. The tetramer–Ptr*WOX4a* system (Fig. 7k) is therefore a key regulator of wood formation in *P. trichocarpa*.

Abbreviated cambium proliferation (Fig. 7m) in *OE-*Ptr*VCS2* allows for rapid differentiation and maturation, and thus promotes early secondary xylem formation (Supplementary Text and Supplementary Fig. 5). Expanded cambium proliferation (Fig. 7l) in the ptr*vcs2* ptr*vcs2-h* mutants delays secondary xylem formation (Supplementary Text and Supplementary Fig. 6b). The rate of wood formation is critically important for a tree’s adaptation to biotic and abiotic stresses. To defend against pathogen infection (for example, from stem canker pathogens^60^), trees need to rapidly produce more specialized wood, the ‘defence wood’, to limit pathogen growth^61,62^. ‘Tension wood’ is another type of specialized wood formed in trees, particularly in *Populus* and *Eucalyptus*, in response to the perception of gravity or mechanical stresses, such as wind and bending^63–65^. Less wood may allow tree stems or branches to be more flexible so that they can tolerate bending without breakage. This flexibility is a well-known feature of the ‘rubbery wood’ in apple trees^66^. Although the extent to which the tetramer–Ptr*WOX4a* system could affect the plasticity of wood remains to be explored, the system is probably specific to forest trees or to *Populus*.

In addition to the abbreviated cambium zone, *OE*-Ptr*VCS2* and *OE-*Ptr*VCS2-h* exhibited severely retarded growth (Fig. 2a and Extended Data Fig. 1b,f). In WT shoot apices, or the primary growth stage (internodes 1 to 4), the expression of Ptr*VCS2* and Ptr*VCS2-h* was negligible compared with that in cambium of the secondary growth stage (represented by internodes 20 and 40) (Extended Data Fig. 2d). The low expression levels of Ptr*VCS2* and Ptr*VCS2-h* in shoot apices of the WT and in the ptr*vcs2* ptr*vcs2-h* mutant having no effects on plant growth (Extended Data Fig. 2d and Fig. 2e) indicate that these two Ptr*VCS2*s have no direct regulatory function in plant primary growth. We also found that cambium cell-layer abatement occurred in the internodes of the *OE*-Ptr*VCS2* and *OE-*Ptr*VCS2-h* transgenics when compared with those of the WT after the same growth or stem elongation period (30 days; Supplementary Fig. 3). These results excluded the effects of the developmental age between the WT plants and the transgenics on secondary vascular growth and supported the notion that high expression levels of Ptr*VCS2* or Ptr*VCS2-h* resulted in fewer cambium cell layers and thus reduced secondary development of stems. However, it is still possible that the primary growth deficits in the overexpression lines affect the secondary growth. We suggest that the strong 35S-promoter-driven ectopic expression of Ptr*VCS2* or Ptr*VCS2-h* may adversely interfere with the normal SAM system, broadly nullifying growth signalling pathways and regulation from the primary to the secondary growth, thereby reducing the growth rate. These suggestions need to be tested and verified—for example, by conducting reciprocal grafting experiments between the WT plants and the transgenics, which may also lead to new insights into the transition from primary to secondary growth. Such insights are particularly important for forest tree species, where this transition could have profound impacts on woody biomass production. Our work provides unique biological resources (transgenics and mutant trees) that could help shed light on the complex co-regulation of growth and adaptation during wood formation. These resources also represent vascular cambium systems for understanding lateral meristem development—that is, its stem cells and their differentiation, specifically for wood formation.

## Methods

### Plant materials and growth conditions

*P. trichocarpa* Torr. & Gray (genotype Nisqually-1) was used for all experiments. WT, transgenic and mutant plants were grown in a greenhouse under controlled environmental conditions (21 to 25 °C, 16 h light/8 h dark and 60–80% humidity)^48^. Stems of healthy 4-month-old clonally propagated *P*. *trichocarpa* plants were used for paraffin sections, in situ hybridization, RNA extraction, and histochemical and histological analysis. Six-month-old plants were harvested for ChIP assays and for the isolation of SDX protoplasts.

### LCM

The 8th stem internodes of *P. trichocarpa* were cut into 2 mm segments and fixed with 100% acetone for a total of 1 h under vacuum at room temperature. After vacuum treatment, the segments were fixed with 100% acetone at 4 °C overnight and then fixed at 37 °C for 1 h. The fixed segments were dehydrated in a graded *n*-butanol:acetone series (30:70, 50:50, 70:30 and 90:10; v/v) and 100% *n*-butanol at 58 °C. The segments were then immersed in paraffin:*n*-butanol solution (50:50, 60:40 and 80:20; v/v) and 100% paraffin (Sigma, P3683) sequentially at 58 °C. The embedded segments were sectioned into 16 µm by a rotary microtome (Leica RM2245). The sections were attached to a nuclease-free frame slide with PET membranes (Leica, 11505190) and then dewaxed with 100% xylene for 4 min. The tissue sections were totally air-dried at room temperature before use. The developing cambium, differentiating xylem and developing phloem cells from the prepared tissue sections were dissected by a laser microscope (Leica Laser Microdissection Microscope, LMD7000)^65^ and collected into RNeasy Lysis Buffer (Qiagen, RNeasy Plant Mini Kit, 74904).

### Total RNA extraction and RNA amplification

An RNeasy Plant Mini Kit (Qiagen, 74904) was used to isolate total RNA from cambium, LCM-collected samples and SDX protoplasts. RNA concentration was detected with a NanoDrop 2000 spectrophotometer (Thermo Scientific), and RNA quality and integrity were analysed with an Agilent 2100 Bioanalyzer (Agilent). For the RNA amplification of LCM-based samples, 750 pg of RNA was used as the starting RNA^65^. The amplification was performed with the Ovation RNA-Seq System V2 Kit (NuGEN, 7102) as described in the manufacturer’s handbook.

### RNA-seq and data analysis

To identify VCS TF genes, RNA-seq was performed with total RNA isolated from developing cambium, differentiating xylem and developing phloem cells collected from *P. trichocarpa* stems by LCM. A total of nine RNA-seq libraries for three biological replicates were generated using a TruSeq RNA Library Prep Kit (Illumina, RS-122-9001DOC), followed by sequencing with the Illumina HiSeq 4000 platform to obtain paired-end reads with a length of 150 base pairs (bp). To detect gene expression in *OE*-Ptr*VCS2* transgenic lines, cambium cell mixture was collected by scraping slightly on the inner side of bark peeled from the WT and transgenic *P. trichocarpa* stems using a double-edged razor blade. A total of six RNA-seq libraries for three biological replicates were generated using a NEBNext Ultra RNA Library Prep Kit (NEB, 7530), followed by sequencing with the Illumina HiSeq × Ten system to obtain paired-end reads with a length of 150 bp. After the sequencing data were filtered with SOAPnuke^67^, the clean reads were aligned to the *P. trichocarpa* genome v.3.0 (Phytozome) by using Bowtie2 (ref. 68). The raw counts were determined and normalized following our established analysis pipeline^42^. DEGs were characterized by FDR < 0.05 by using DESeq2 (ref. 69).

### RT–qPCR

RT reactions were performed using TaqMan Reverse Transcription Reagents (Invitrogen, N8080234) following the manufacturer’s protocol. All qPCRs were carried out on the Agilent M×3000P Real-Time PCR System with FastStart Universal SYBR Green Master Mix (Roche, 4913914001) following the standard protocol. Gene expression was normalized to the expression of the Ptr*ACTIN* gene. The primers used for RT–qPCR and ChIP–qPCR are listed in Supplementary Table 6.

### Generation of gene overexpression and CRISPR-edited transgenic *P. trichocarpa*

The coding sequences of Ptr*VCS2*, Ptr*VC2-h* and Ptr*WOX4a* were amplified from the complementary DNA prepared from the cambium of *P. trichocarpa* plants, followed by assembling the coding sequences into the pBI121 vector driven by a CaMV 35S promoter for generating overexpression constructs. The CRISPR–Cas9 system^70^ was used to generate single-knockout mutants of Ptr*VCS2* and double-knockout mutants of Ptr*VCS2* and Ptr*VCS2-h*, Ptr*WOX4a* and Ptr*WOX4b*, and Ptr*GCN5-1* and Ptr*GCN5-2*. The single guide RNAs designed by CRISPR-P v.2.0 (http://crispr.hzau.edu.cn/cgi-bin/CRISPR2/CRISPR) were synthesized and cloned into the pMgP237–2A–GFP vector for targeting Ptr*VCS2* and Ptr*VCS2-h* or the pEgP237–2A–GFP vector for targeting Ptr*WOX4a* and Ptr*WOX4b*, and Ptr*GCN5-1* and Ptr*GCN5-2*. All transgenic plants were generated by *Agrobacterium tumefaciens*–mediated transformation of *P. trichocarpa*^65^. The *A. tumefaciens* cells (GV3101) were cultured to OD_600_ of 0.4 and infected with stem explants from healthy tissue-cultured *P. trichocarpa* seedlings. After regeneration, the transgenic plants were confirmed by genomic DNA PCR analysis. The expression levels of the transgenes in the cambium of the transgenic plants were detected by RT–qPCR. For identification of the knockout mutants, the genome sequences containing the single guide RNA target sites were amplified from the genome DNA prepared from mutant plants and sequenced in the pMD18-T vector (Takara, 6011). At least 20 colonies were selected for sequencing. The primers for vector construction, RT–qPCR and mutation detection are listed in Supplementary Table 6.

### RNA in situ hybridization

The 6th stem internodes of *P. trichocarpa* were cut into 2 mm segments and fixed in formalin–acetic acid–alcohol liquid solution (50% ethanol, 5% acetic acid and 3.7% formaldehyde; v/v) overnight at 4 °C. The fixed segments were dehydrated in a graded ethanol series at 4 °C and then incubated in 50% xylene in ethanol and 100% xylene at room temperature. After dehydration, the fixed segments were embedded in paraffin (Sigma, P3683) and sectioned into 12 µm by a rotary microtome (Leica RM2245). The tissue sections were then attached to a poly-l-lysine-coated glass slide (Sigma, P0425) for hybridization. A 207-bp region of Ptr*VCS2* and a 187-bp region of Ptr*WOX4a* were selected as specific probes. The antisense and sense probes were synthesized with T7 RNA polymerase and labelled using a digoxigenin RNA labelling kit (Roche, 11175025910). The in vitro transcription reactions and quantification were performed following the manufacturer’s protocol. After deparaffinization and pretreatment, the tissue sections were incubated with 250 ng ml^−1^ digoxigenin-labelled antisense or sense probes at 48 °C overnight in the hybridization solution containing 50% (v/v) formamide. After hybridization, a digoxigenin nucleic acid detection kit (Roche, 11175041910) was used for the detection of digoxigenin-labelled probes and colour reactions following the manufacturer’s instructions. Before colour observation, the slides were rinsed in 70% (v/v) ethanol for 2 min once, 100% ethanol for 2 min twice and xylene for 1 min, and then sealed with neutral balsam (Solarbio, G8590). The images were captured by a digital microscope and scanner M8 (Precipoint).

### Histochemical and histological analysis

The internodes of the same age (30-day growth) were prepared by marking the newborn internodes of the WT and transgenic plants at the same date and collecting the marked internodes after 30 days. Histochemical and histological analyses were performed as described previously^71^. Briefly, stem internodes of *P. trichocarpa* were cut into 2 mm segments and fixed with formalin–acetic acid–alcohol solution. The fixed segments were dehydrated in a graded ethanol series at 4 °C and then incubated in 50% xylene in ethanol and 100% xylene at room temperature. For paraffin embedding, the dehydrated segments were incubated in xylene/paraffin (75:25; v/v) overnight at 42 °C and in 100% paraffin (Sigma, P3683) at 60 °C. The embedded fragments were sectioned into 12 µm by using a rotary microtome (Leica RM2245). The sections were stained with toluidine blue O or with safranin O and fast green. Stem section micrographs were processed using a scanner M8 (Precipoint) and ViewPoint (v.1.0.0.0, PreciPoint, Freising, Germany) setup software.

### Gene expression analysis in SDX protoplasts

The coding sequences of Ptr*VCS2* and Ptr*WOX13a* were inserted into the pENTR/D-TOPO vector (Invitrogen, 450218), followed by recombining into the pUC19–*35S*_*pro*_–*RfA*–*35S*_*pro*_–*sGFP*^72^ destination vector, generating pUC19–*35S*_*pro*_–Ptr*VCS2*–*35S*_*pro*_–*sGFP* and pUC19–*35S*_*pro*_–Ptr*WOX13a*–*35S*_*pro*_–*sGFP*. Using a CsCl gradient, the plasmids were extracted and transfected into SDX protoplasts following an established protocol^42,43^. The transfected protoplasts were cultured for 12 h and collected for RNA extraction and RT–qPCR analysis as described above. For the glucocorticoid receptor (GR)-based inducible system^47^, the coding sequence of Ptr*WOX13a* was fused with the GR domain and cloned into the p2GW7 vector^47,73^, generating the p2GW7–*35S*_*pro*_–Ptr*WOX13a*–*GR* effector construct. The promoter sequence of Ptr*WOX4a* (2,032 bp upstream of ATG) was amplified from the *P*. *trichocarpa* genome and inserted into the pGreen0800 vector^74^, generating the pGreen0800–Ptr*WOX4a*_*pro*_–*LUC* reporter construct. *P*. *trichocarpa* SDX protoplasts were transfected with the effector and reporter constructs as described above. The transfected protoplasts were cultured for 12 h and were then treated with 10 µM dexamethasone (Sigma, D4902) in ethanol for 6 h to allow PtrWOX13a–GR to translocate into the nuclei. The same amount of ethanol alone was used for treating the protoplasts as a control. To block new protein synthesis, 2 µM cycloheximide (CHX, MCE, HY-12320) in DMSO or DMSO alone (as a control) was applied to the protoplasts for 30 min before the addition of the dexamethasone. The treated protoplasts were then used for dual-luciferase reporter activity assays with a kit (Promega, E1910) according to the manufacturer’s protocol or RT–qPCR analysis as described above. Three biological replicates were performed. The primers for construct generation and RT–qPCR are listed in Supplementary Table 6.

### ChIP assays

The cambium from *P. trichocarpa* stems was harvested for the ChIP assays following our established protocol^59^. Anti-FLAG (Sigma, F1804, 5 μg ml^−1^), anti-H3K9ac (Abcam, ab10812, 5 μg ml^−1^), anti-H3K14ac (Abcam, ab52946, 5 μg ml^−1^), anti-H3K27ac (Abcam, ab4729, 5 μg ml^−1^), anti-PtrVCS2 (Abmart, 6 μg ml^−1^) or anti-IgG (Abcam, ab205719, 5 μg ml^−1^) antibodies were used for immunoprecipitation of the fragmented chromatin. The anti-PtrVCS2 monoclonal antibody (against the full-length PtrVCS2 protein) was produced in mice and purified by the IgG affinity chromatography column (Abmart). After immunoprecipitation, the ChIP–DNA was purified and quantified using Qubit Fluorometer. The ChIP–DNA was used for ChIP–qPCR analysis or ChIP-seq library construction. The primers used for ChIP–qPCR are listed in Supplementary Table 6. For ChIP-seq library construction, six libraries (ChIP–DNA and input DNA for three biological replicates) were produced by using the NEBNext Multiplex Oligos for Illumina (NEB, E7335S) and the NEBNext Ultra II DNA Library Prep Kit for Illumina (NEB, E7645S) following the manufacturer’s protocol. The ChIP-seq libraries were sequenced using an Illumina NextSeq 500 platform.

### ChIP-seq data analysis

Single-end reads with an average length of 50 bp were obtained. The sequencing reads were processed to trim adaptor sequences and filter low-quality reads using FASTX-Toolkit (v.0.0.14) (http://hannonlab.cshl.edu/fastx_toolkit/). The processed reads were mapped to the *P. trichocarpa* genome reference v.3.0 using Bowtie 2 (v.2.3.5.1) allowing for no more than one mismatch^68^. After duplicated reads were removed, uniquely mapped reads were used for peak identification, using MACS2 (ref. 75) with the default parameters (*P* < 1 × 10^−5^). Peaks identified in two or three biological replications (peak summits between replications were less than 100 bp) were defined as PtrVCS2 binding peaks. Each peak was assigned to the closest gene. Genes containing one or more PtrVCS2 binding peaks within the 3-kb promoter region were defined as PtrVCS2 target genes. For the discovery of binding motifs, 500-bp flanking sequences around the peak summits of PtrVCS2 were used for MEME-ChIP^76^ (Multiple Em for Motif Elicitation) analysis with Fisher’s exact test.

### Y2H assays

Y2H assays were carried out according to the Matchmaker Gold Yeast Two-Hybrid System (Clontech, 630489). The full-length coding region of Ptr*VCS2* was cloned into the GAL4 binding domain vector (pGBKT7, Clontech, 630489). A total of 59 VCS TFs (Supplementary Table 4) were fused to the GAL4 activating domain in the pGADT7 vector (Clontech, 630489), thereby generating a collection of 59 VCS TFs for Y2H screening. The transformed yeasts (strain Y2HGold) with the binding domain and activating domain constructs were incubated on selection medium −LW (SD/−Leu/−Trp) and −LWH/X (SD/−Leu/−Trp/−His/X-α-Gal) with 40 mg ml^−1^ X-α-Gal to assess their growth status.

### BiFC assays

The coding regions of Ptr*VCS2*, Ptr*WOX13a*, Ptr*VCS19*, Ptr*VCS94*, Ptr*WOX4b*, Ptr*GCN5-1* and Ptr*ADA2b-3* were cloned into the pUGW0 and pUGW2 vectors. Each pair of the constructs (Ptr*VCS2*–*YFP*^*N*^/Ptr*WOX13a*–*YFP*^*C*^, Ptr*VCS2*–*YFP*^*N*^/Ptr*VCS19*–*YFP*^*C*^, Ptr*VCS2*–*YFP*^*N*^/Ptr*VCS94*–*YFP*^*C*^, Ptr*VCS2*–*YFP*^*N*^/Ptr*WOX4b*–*YFP*^*C*^, Ptr*VCS2*–*YFP*^*N*^/Ptr*GCN5-1*–*YFP*^*C*^, Ptr*GCN5-1*–*YFP*^*N*^/Ptr*WOX13a*–*YFP*^*C*^, Ptr*ADA2b-3*–*YFP*^*N*^/Ptr*WOX13a*–*YFP*^*C*^, Ptr*ADA2b-3*–*YFP*^*N*^/Ptr*VCS2*–*YFP*^*C*^, Ptr*ADA2b-3*–*YFP*^*N*^/Ptr*GCN5-1*–*YFP*^*C*^ and Ptr*GCN5-1*–*YFP*^*C*^/Ptr*AREB1-2*–*YFP*^*N*^) was co-transfected into *P. trichocarpa* SDX protoplasts with the *H2A-1*–*mCherry* nuclear marker or Ptr*VCS2*–*mCherry* following an established protocol^43^. The *YFP*^*C*^ or *YFP*^*N*^ empty vector was used as a negative control. The transfected protoplasts were cultured for 12 h and were then collected for observation by using a confocal laser scanning microscope (Zeiss LSM 800). Quantification of the YFP fluorescence signals was carried out using ImageJ (1.53e)^77^. The average local background signal (measured in the region without a cell) was subtracted from the values. Each transformation was performed with three biological replicates, and more than 30 individual protoplast cells with specific fluorescent signals were measured.

### Expression and purification of recombinant proteins

The full-length coding sequences of Ptr*VCS2*, Ptr*WOX13a*, Ptr*ZHD1*, Ptr*GCN5-1* and Ptr*ADA2b-3* were inserted into the pET101/D-TOPO vector (Invitrogen, K10101) for generating the 6×His tag fusion proteins. The primers used for construct generation are listed in Supplementary Table 6. Recombinant proteins were expressed in the *Escherichia coli* BL21 strain, followed by purification with HisPur Ni-NTA Resin (Thermo Scientific, 88221). After washing and elution, the recombinant proteins were collected in PBS buffer (137 mM NaCl, 2.7 mM KCl, 10 mM Na_2_HPO_4_, 2 mM KH_2_PO_4_) using Centrifugal Filter Devices (Millipore, UFC501096).

### Pull-down assays

For the pull-down assays, the pETDuet-1 vector (Novagen, 71146) was used to produce recombinant proteins fused with 6×His tag and recombinant proteins fused with S tag. The coding sequences of Ptr*VCS2*, Ptr*WOX13a*, Ptr*GCN5-1*, Ptr*ADA2b-3* and *GFP* were cloned into the pETDuet-1 vector to generate constructs harbouring, respectively, Ptr*VCS2–6×His-tag* and Ptr*WOX13a–S-tag*, Ptr*GCN5-1–6×His-tag* and Ptr*VCS2–S-tag*, Ptr*GCN5-1–6×His-tag* and Ptr*WOX13a–S-tag*, Ptr*WOX13a–6×His-tag* and Ptr*ADA2b-3–S-tag*, Ptr*WOX13a–6×His-tag* and Ptr*VCS2–S-tag*, Ptr*VCS2–6×His-tag* and *GFP–S-tag*, Ptr*GCN5-1–6×His-tag* and *GFP–S-tag*, and Ptr*WOX13a–6×His-tag* and *GFP–S-tag*. *GFP* was used as a negative control. For FLAG-tagged protein constructs, we cloned the coding sequences of Ptr*WOX13a*-fused *3×FLAG*, Ptr*ADA2b-3*-fused *3×FLAG* and *GUS*-fused *3×FLAG* into the *Nde*I–*Pac*I-digested pETDuet-1 vector, respectively, to generate single-tagged Ptr*WOX13a–3×FLAG*, Ptr*ADA2b-3–3×FLAG* and *GUS–3×FLAG* constructs. *GUS–3×FLAG* was used as a negative control. The primers used for construct generation are listed in Supplementary Table 6. For dimer or trimer pull-down, the 6×His-tagged proteins were used as bait proteins, and S-tagged or 3×FLAG-tagged proteins were used as prey proteins. The recombinant bait proteins together with the prey proteins were incubated with HisPur Ni-NTA Resin (Thermo Scientific, 88221) in binding buffer (50 mM NaH_2_PO_4_, pH 8.0, 500 mM NaCl) for 2 h at 4 °C, followed by washing the beads with 20 mM imidazole in washing buffer and eluting the proteins with 250 mM imidazole in elution buffer. The pulled-down proteins were analysed by SDS–PAGE and detected by immunoblotting using anti-His (Abcam, ab1187), anti-S (Abcam, ab183674) and anti-FLAG (Sigma, F1804) antibodies.

### Immunoblotting

SDS–PAGE electrophoresis was used to separate proteins. The protein samples were then transferred to a PVDF membrane (Millipore, IPVH00010). After blocking with non-fat dry milk, the membranes were probed with the corresponding antibodies (anti-His antibody, Abcam, ab1187, 1:10,000 dilution; anti-S antibody, Abcam, ab183674, 1:10,000 dilution; anti-FLAG antibody, Sigma, F1804, 1:2,000 dilution) at 4 °C overnight. Signals were detected by using SuperSignal West Pico Chemiluminescent Substrate (Thermo Scientific, 34577) and X-ray film (Sigma, F1274-50EA).

### EMSA

The PtrWOX13a, PtrVCS2 and PtrZHD1 recombinant proteins were produced from *E. coli* as described above. The PtrZHD1 recombinant protein and an empty pET101/D-TOPO vector were used as negative controls. DNA probes were synthesized and labelled with biotin at the 3′ end (Thermo Scientific, 89818). The CAATCAC sequences in the promoter fragments were mutated by changing the third A to T to generate mutated probes. The primers used for probe preparation are listed in Supplementary Table 6. EMSAs were carried out following the manufacturer’s protocol with the Lightshift Chemiluminescent EMSA kit (Thermo Scientific, 20148). Briefly, binding reactions were performed by incubating the probes and recombinant proteins for 20 min at room temperature in binding buffer (10 mM Tris–HCl, pH 7.5, 50 mM KCl, 10 mM EDTA, 2.5% glycerol (v/v), 5 mM MgCl_2_, 0.05% Nonidet P-40 (v/v), and 50 ng µl^−1^ poly (dI-dC)). Unlabelled WT or mutated probes (100-, 200- or 400-fold of labelled probes) were used as competitors. Protein–DNA mixtures were separated on a 6% (w/v) nondenaturing polyacrylamide gel and transferred to a nylon membrane (Thermo Scientific, 77016). After the transferred protein–DNA mixtures were crosslinked with the membrane, the biotin-labelled DNA was detected with chemiluminescence.

### HAT activity assays

The recombinant proteins PtrVCS2, PtrGCN5-1 and PtrADA2b-3 were produced from *E. coli* as described above. The HAT assays were carried out using the HAT Fluorometric Assay Kit (BioVision, K334-100) following the manufacturer’s instructions. Briefly, the purified recombinant proteins were added to the HAT Assay Buffer. HeLa Nuclear Extract was used as the positive control protein. The HAT Assay Buffer was used as a background control. The enzymatic activity was measured by a fluorescence plate reader in kinetic mode at 25 °C for 40 min (excitation/emission, 535/587 nm).

### Statistical analysis

Two-tailed Student’s *t*-tests were carried out for all statistical analyses to determine significance. Significance levels were defined as *P < 0.05, **P < 0.01 and ***P < 0.001.

### Reporting summary

Further information on research design is available in the Nature Portfolio Reporting Summary linked to this article.

## Supplementary information

Supplementary Information Supplementary Text and Figs. 1–11.

Reporting Summary

Supplementary Tables Supplementary Tables 1–6.

Supplementary Data Statistical source data for supplementary figures.

## Data Availability

The data supporting the findings of this study are available in the article and its Supplementary Information files. The RNA-seq and ChIP-seq data have been deposited in the National Center for Biotechnology Information Sequence Read Archive under accession numbers SRR18274403–SRR18274417 and SRR18272729–SRR18272734. Sequence data from this article can be found in *P. trichocarpa* genome v.3.0 (Phytozome, https://phytozome.jgi.doe.gov/pz/portal.html) under the accession numbers listed in Supplementary Tables 1 and 6. Source data are provided with this paper.

## Source data

Source Data Fig. 2 Statistical source data.
Source Data Fig. 3 Statistical source data.
Source Data Fig. 4 Statistical source data.
Source Data Fig. 5 Statistical source data and unprocessed western blots.
Source Data Fig. 6 Statistical source data and unprocessed western blots.
Source Data Fig. 7 Statistical source data.
Source Data Extended Data Fig. 1 Statistical source data.
Source Data Extended Data Fig. 2 Statistical source data.
Source Data Extended Data Fig. 3 Statistical source data.
Source Data Extended Data Fig. 4 Statistical source data.
Source Data Extended Data Fig. 5 Statistical source data.
Source Data Extended Data Fig. 8 Unprocessed western blots.
Source Data Extended Data Fig. 10 Statistical source data.

## References

[1] Esau, K. Vascular Differentiation in Plants (Holt, Rinehart, & Winston, 1965).

[2] Evert, R. F. & Eichhorn, S. E. Esau’s Plant Anatomy: Meristems, Cells, and Tissues of the Plant Body—Their Structure, Function, and Development 3rd edn (John Wiley & Sons, 2006).

[3] Timell TE (1980). Organization and ultrastructure of the dormant cambial zone in compression wood of Picea abies. Wood Sci. Technol..

[4] Wloch W (1981). Nonparallelism of cambium cells in neighboring rows. Acta Soc. Bot. Pol..

[5] Mahmood A (1968). Cell grouping and primary wall generations in the cambial zone, xylem, and phloem in Pinus. Aust. J. Bot..

[6] Evert RF, Deshpande BP (1970). An ultrastructural study of cell division in the cambium. Am. J. Bot..

[7] Sanio K (1873). Anatomie der gemeinen Kiefer (Pinus sylvestris L.). Jahrb. Wiss. Bot..

[8] Srivastava LM, O’Brien TP (1966). On the ultrastructure of cambium and its vascular derivatives. Protoplasma.

[9] Murmanis L (1970). Locating the initial in the vascular cambium of Pinus strobus L. by electron microscopy. Wood Sci. Technol..

[10] Srivastava LM (1966). On the fine structure of the cambium of Fraxinus americana L. J. Cell Biol..

[11] Isebrands JG, Larson PR (1973). Some observations on the cambial zone in cottonwood. Int. Assoc. Wood Anat..

[12] Murmanis L (1977). Development of vascular cambium into secondary tissue of Quercus rubra L. Ann. Bot..

[13] Baïer M (1994). Pectin changes in samples containing poplar cambium and inner bark in relation to the seasonal cycle. Planta.

[14] Larson, P. R. The Vascular Cambium: Development and Structure (Springer, 1994).

[15] Aichinger E, Kornet N, Friedrich T, Laux T (2012). Plant stem cell niches. Annu. Rev. Plant Biol..

[16] Mayer KF (1998). Role of WUSCHEL in regulating stem cell fate in the Arabidopsis shoot meristem. Cell.

[17] Sarkar AK (2007). Conserved factors regulate signalling in Arabidopsis thaliana shoot and root stem cell organizers. Nature.

[18] Schoof H (2000). The stem cell population of Arabidopsis shoot meristems is maintained by a regulatory loop between the CLAVATA and WUSCHEL genes. Cell.

[19] Stahl Y, Wink RH, Ingram GC, Simon R (2009). A signaling module controlling the stem cell niche in Arabidopsis root meristems. Curr. Biol..

[20] Servet C, Conde e, Silva N, Zhou DX (2010). Histone acetyltransferase AtGCN5/HAG1 is a versatile regulator of developmental and inducible gene expression in Arabidopsis. Mol. Plant.

[21] Bertrand C, Bergounioux C, Domenichini S, Delarue M, Zhou D-X (2003). Arabidopsis histone acetyltransferase AtGCN5 regulates the floral meristem activity through the WUSCHEL/AGAMOUS pathway. J. Biol. Chem..

[22] Bollier N (2018). At-MINI ZINC FINGER2 and Sl-INHIBITOR OF MERISTEM ACTIVITY, a conserved missing link in the regulation of floral meristem termination in Arabidopsis and tomato. Plant Cell.

[23] Zhang Y, Jiao Y, Liu Z, Zhu Y-X (2015). ROW1 maintains quiescent centre identity by confining WOX5 expression to specific cells. Nat. Commun..

[24] Etchells JP, Provost CM, Mishra L, Turner SR (2013). WOX4 and WOX14 act downstream of the PXY receptor kinase to regulate plant vascular proliferation independently of any role in vascular organisation. Development.

[25] Zhang J (2019). Transcriptional regulatory framework for vascular cambium development in Arabidopsis roots. Nat. Plants.

[26] Hirakawa Y (2008). Non-cell-autonomous control of vascular stem cell fate by a CLE peptide/receptor system. Proc. Natl Acad. Sci. USA.

[27] Hirakawa Y, Kondo Y, Fukuda H (2010). TDIF peptide signaling regulates vascular stem cell proliferation via the WOX4 homeobox gene in Arabidopsis. Plant Cell.

[28] Wang D (2021). Vascular cambium: the source of wood formation. Front. Plant Sci..

[29] Fischer U, Kucukoglu M, Helariutta Y, Bhalerao RP (2019). The dynamics of cambial stem cell activity. Annu. Rev. Plant Biol..

[30] Kucukoglu M, Nilsson J, Zheng B, Chaabouni S, Nilsson O (2017). WUSCHEL-RELATED HOMEOBOX4 (WOX4)-like genes regulate cambial cell division activity and secondary growth in Populus trees. N. Phytol..

[31] Zhu Y, Song D, Xu P, Sun J, Li L (2018). A HD-ZIP III gene, PtrHB4, is required for interfascicular cambium development in Populus. Plant Biotechnol. J..

[32] Zhu Y, Song D, Sun J, Wang X, Li L (2013). PtrHB7, a class III HD-Zip gene, plays a critical role in regulation of vascular cambium differentiation in Populus. Mol. Plant.

[33] Robischon M, Du J, Miura E, Groover A (2011). The Populus class III HD ZIP, popREVOLUTA, influences cambium initiation and patterning of woody stems. Plant Physiol..

[34] Zheng S (2020). Two MADS-box genes regulate vascular cambium activity and secondary growth by modulating auxin homeostasis in Populus. Plant Commun..

[35] Du J, Mansfield SD, Groover AT (2009). The Populus homeobox gene ARBORKNOX2 regulates cell differentiation during secondary growth. Plant J..

[36] Du J, Miura E, Robischon M, Martinez C, Groover A (2011). The Populus Class III HD ZIP transcription factor POPCORONA affects cell differentiation during secondary growth of woody stems. PLoS ONE.

[37] Hou J (2020). MiR319a-targeted PtoTCP20 regulates secondary growth via interactions with PtoWOX4 and PtoWND6 in Populus tomentosa. N. Phytol..

[38] Mackay JP, Crossley M (1998). Zinc fingers are sticking together. Trends Biochem. Sci..

[39] Takatsuji H (1999). Zinc-finger proteins: the classical zinc finger emerges in contemporary plant science. Plant Mol. Biol..

[40] Liu M (2019). Genome-wide investigation of the ZF-HD gene family in Tartary buckwheat (Fagopyrum tataricum). BMC Plant Biol..

[41] Hu W, Ma H (2006). Characterization of a novel putative zinc finger gene MIF1: involvement in multiple hormonal regulation of Arabidopsis development. Plant J..

[42] Lin YC (2013). SND1 transcription factor-directed quantitative functional hierarchical genetic regulatory network in wood formation in Populus trichocarpa. Plant Cell.

[43] Lin YC (2014). A simple improved-throughput xylem protoplast system for studying wood formation. Nat. Protoc..

[44] Liu B (2014). WUSCHEL-related Homeobox genes in Populus tomentosa: diversified expression patterns and a functional similarity in adventitious root formation. BMC Genomics.

[45] Sundell D (2017). AspWood: high-spatial-resolution transcriptome profiles reveal uncharacterized modularity of wood formation in Populus tremula. Plant Cell.

[46] Franco-Zorrilla JM (2014). DNA-binding specificities of plant transcription factors and their potential to define target genes. Proc. Natl Acad. Sci. USA.

[47] Aoyama T, Chua NH (1997). A glucocorticoid-mediated transcriptional induction system in transgenic plants. Plant J..

[48] Li S (2019). The AREB1 transcription factor influences histone acetylation to regulate drought responses and tolerance in Populus trichocarpa. Plant Cell.

[49] Brownell JE (1996). Tetrahymena histone acetyltransferase A: a homolog to yeast Gcn5p linking histone acetylation to gene activation. Cell.

[50] Grant PA (1997). Yeast Gcn5 functions in two multisubunit complexes to acetylate nucleosomal histones: characterization of an Ada complex and the SAGA (Spt/Ada) complex. Genes Dev..

[51] Balasubramanian R, Pray-Grant MG, Selleck W, Grant PA, Tan S (2002). Role of the Ada2 and Ada3 transcriptional coactivators in histone acetylation. J. Biol. Chem..

[52] Etchells JP, Provost CM, Turner SR (2012). Plant vascular cell division is maintained by an interaction between PXY and ethylene signalling. PLoS Genet..

[53] Brackmann K (2018). Spatial specificity of auxin responses coordinates wood formation. Nat. Commun..

[54] Smetana O (2019). High levels of auxin signalling define the stem-cell organizer of the vascular cambium. Nature.

[55] Fu X (2021). Cytokinin signaling localized in phloem noncell-autonomously regulates cambial activity during secondary growth of Populus stems. N. Phytol..

[56] Hu J (2022). AUXIN RESPONSE FACTOR7 integrates gibberellin and auxin signaling via interactions between DELLA and AUX/IAA proteins to regulate cambial activity in poplar. Plant Cell.

[57] Jaenisch R, Bird A (2003). Epigenetic regulation of gene expression: how the genome integrates intrinsic and environmental signals. Nat. Genet..

[58] Kouzarides T (2007). Chromatin modifications and their function. Cell.

[59] Li W (2014). A robust chromatin immunoprecipitation protocol for studying transcription factor–DNA interactions and histone modifications in wood-forming tissue. Nat. Protoc..

[60] Newcombe G, Ostry M (2001). Recessive resistance to Septoria stem canker of hybrid poplar. Phytopathology.

[61] Shigo AL (1984). Compartmentalization: a conceptual framework for understanding how trees grow and defend themselves. Ann. Rev. Phytopathol..

[62] Dhillon B (2015). Horizontal gene transfer and gene dosage drives adaptation to wood colonization in a tree pathogen. Proc. Natl Acad. Sci. USA.

[63] Scurfield G (1973). Reaction wood: its structure and function: lignification may generate the force active in restoring the trunks of leaning trees to the vertical. Science.

[64] Liu B (2021). Transcriptional reprogramming of xylem cell wall biosynthesis in tension wood. Plant Physiol..

[65] Yu J (2022). A PtrLBD39-mediated transcriptional network regulates tension wood formation in Populus trichocarpa. Plant Commun..

[66] Beakbane AB, Thompson EC (1945). Abnormal lignification in the wood of some apple trees. Nature.

[67] Li R, Li Y, Kristiansen K, Wang J (2008). SOAP: short oligonucleotide alignment program. Bioinformatics.

[68] Langmead B, Salzberg SL (2012). Fast gapped-read alignment with Bowtie 2. Nat. Methods.

[69] Love MI, Huber W, Anders S (2014). Moderated estimation of fold change and dispersion for RNA-seq data with DESeq2. Genome Biol..

[70] Ueta R (2017). Rapid breeding of parthenocarpic tomato plants using CRISPR/Cas9. Sci. Rep..

[71] Wang Z (2020). MYB Transcription factor 161 mediates feedback regulation of secondary wall-associated NAC-Domain1 family genes for wood formation. Plant Physiol..

[72] Li Q (2012). Splice variant of the SND1 transcription factor is a dominant negative of SND1 members and their regulation in Populus trichocarpa. Proc. Natl Acad. Sci. USA.

[73] Huang D (2015). A gibberellin-mediated DELLA–NAC signaling cascade regulates cellulose synthesis in rice. Plant Cell.

[74] Hellens RP (2005). Transient expression vectors for functional genomics, quantification of promoter activity and RNA silencing in plants. Plant Methods.

[75] Zhang Y (2008). Model-based analysis of ChIP-Seq (MACS). Genome Biol..

[76] Machanick P, Bailey TL (2011). MEME-ChIP: motif analysis of large DNA datasets. Bioinformatics.

[77] Schneider CA, Rasband WS, Eliceiri KW (2012). NIH Image to ImageJ: 25 years of image analysis. Nat methods.

