# Supplementary material for: Cell-type-specific PtrWOX4a and PtrVCS2 form a regulatory nexus with a histone modification system for stem cambium development in Populus trichocarpa
Source: Nat Plants. 2023 Jan 9;9(1):96–111. doi: 10.1038/s41477-022-01315-7 (PMC9873556; doi:10.1038/s41477-022-01315-7)
Supplement: Supplementary file 1 — Supplementary Text and Figs. 1–11. [file 41477_2022_1315_MOESM1_ESM.pdf]

nature plants

Article https://doi.org/10.1038/s41477-022-01315-7

Cell-type-specific PtrWOX4a and PtrVCS2 form a regulatory nexus with a histone modification system for stem cambium development in *Populus trichocarpa*

In the format provided by the authors and unedited

Supplementary Text

This supplementary text contains additional descriptive and explanatory information that could not be included in the main text due to space limitations.

1. Identification of vascular cambium-specific transcription factors in *P. trichocarpa*

We used laser capture microdissection (LCM) to isolate developing cambium (C), differentiating xylem (X), and developing phloem (P) cells from the stem of *P. trichocarpa* (Fig. 1a,b and Methods) for full transcriptome RNA-seq analysis (Methods). We thus identified 95 "vascular cambium-specific" (VCS) transcription factor (TF) genes from the overlap between 199 TFs whose developing cambium/developing phloem (C/P) transcript abundance ratio was greater than 2 (FDR < 0.05) and 143 TFs whose developing cambium/differentiating xylem (C/X) transcript abundance ratio was greater than 2 (FDR < 0.05). These 95 genes belong to 30 TF families (Supplementary Table 1) and several of their orthologs in other species have been suggested or demonstrated to play roles in regulating cambium activity[1-5]. We named these TF genes *PtrVCS1* to *PtrVCS95* (Supplementary Table 1) based on decreasing transcript levels in the vascular cambium. *PtrVCS1* (Potri.014G025300) is identical to *PtrWOX4a*[6], and is the *P. trichocarpa* ortholog of the *WUSCHEL-RELATED HOMEOBOX4* (*WOX4*) that plays crucial roles in the maintenance of the vascular cambium in *Arabidopsis*[1,7,8] and *Populus*[6]. *PtrVCS2* (Potri.004G126600), the gene with the second most abundant transcripts, is not related to *PtrVCS1* and encodes a putative zinc finger (ZF) protein that belongs to a putative TF subfamily[9-11] of zinc finger-homeodomain (ZF-HD) proteins[12]. *In situ* mRNA hybridization validated the

tissue specificity of *PtrWOX4a* and *PtrVCS2* in cambium cells (Supplementary Fig. 1 and Fig. 3d). Additionally, we compared the expression profiles obtained for *VCS* genes and the high-spatial-resolution transcriptome profiles of the secondary phloem, vascular cambium, and wood-forming tissues of *P. tremula*[13]. Among 1,869 *VCS* genes, 88% of their homologs in *P. tremula* (1,637 out of 1,869; Supplemental Fig. 2a), including 82 *VCS* TF genes (Supplemental Fig. 2b), were highly expressed in *P. tremula* cambium samples[13]. We also compared the cambium-specific expression profiles to the transcriptome profiles of the shoot tip, young leaf, and primary root tissues for *P. trichocarpa*[14] and determined that 45 *VCS* TF genes (Supplemental Fig. 2c) are specifically expressed in the cambium, including *PtrVCS1* (*PtrWOX4a*) and *PtrVCS2*. In this study, we focused on the two most abundant *VCS* TF genes, *PtrWOX4a* and *PtrVCS2*.

2. Phenotype of *OE-PtrVCS2* transgenics and *ptrvcs2/ptrvcs2-h* double mutants

Serial cross-section analysis of the stem from the 5th to the 20th internodes revealed that vascular cambium of *OE-PtrVCS2* and *OE-PtrVCS2-h* in all internodes examined is devoid of a fixed number (4 to 6) of cambium cell layers compared to the wild-type (WT) (Fig. 2c,d and Extended Data Fig. 1c,d,h-j). To exclude the possible effects of developmental age between the WT and the transgenic plants on secondary vascular growth, we compared the cambium phenotypes of internodes after the same growth or stem elongation period (30 days). The results showed that the number of cambium cell layers was significantly reduced in the *OE-PtrVCS2* (lines *#2, #3*) and *OE-*

PtrVCS2-h (lines *#2, #3*) transgenic lines compared to that in WT (Supplementary Fig. 3). In *Populus*, stem secondary growth (forming wood) normally starts from the 5th internode[15], when a continuous cylindrical vascular cambium is established[16,17]. Overexpression of *PtrVCS2* or *PtrVCS2-h* induced an alteration in three vascular developmental phases. First, in *OE-PtrVCS2* and *OE-PtrVCS2-h*, a cylindrical vascular cambium had already begun to develop in the 1st internode (Supplementary Fig. 4a,b). Second, differentiated or lignified secondary xylem was also already established in the 1st internode (Supplementary Fig. 5a,b), which would normally take place in the 6th internode in WT (Supplementary Fig. 5b). Third, the development of secondary phloem became obvious in the 5th internode in *OE-PtrVCS2* and the 7th internode in *OE-PtrVCS2-h* instead of the 12th internode in WT (Supplementary Fig. 5b).

We generated single and double knockout mutants (Fig. 2e,f and Extended Data Fig. 3a,d) in *PtrVCS2* and *PtrVCS2-h* in *P. trichocarpa* using a CRISPR-Cas9 genome-editing system. We obtained two independent biallelic single mutants (*ptrvcs2 #1* and *ptrvcs2 #2*) and three independent biallelic double mutants (*ptrvcs2/ptrvcs2-h #1*, *ptrvcs2/ptrvcs2-h #2*, and *ptrvcs2/ptrvcs2-h #3*) with insertions and/or deletions that led to frame shifts and premature stop codons (Extended Data Fig. 3a,d). Growth and stem cross-section analyses revealed that the single mutants and the WT exhibited similar growth in plant height (Fig. 2e), stem diameter (Fig. 2f), proliferation in cambium cells (Extended Data Fig. 3b,c), and secondary phloem and secondary xylem development (Supplementary Fig. 6a). Although similar in height to WT plants,

ptrvcs2/ptrvcs2-h double mutants developed stem vascular features that were distinct from the WT and opposite to those of the overexpression lines (*OE-PtrVCS2* or *OE-PtrVCS2-h*). The double mutants had 2 to 4 more cambium cell layers as compared to the WT (the 5th-8th internodes in Fig. 2g,h and Extended Data Fig. 3e,f; the same age internodes [30 days old] in Supplementary Fig. 3), slightly delayed secondary phloem and secondary xylem developments (Supplementary Fig. 6b), and increased stem diameter (Supplementary Fig. 7). These phenotypes observed in the double mutants but not the single mutants confirmed the suggestion that *PtrVCS2* and *PtrVCS2-h* are functionally redundant.

3. RNA-seq analysis of *OE-PtrVCS2*

To investigate how *PtrVCS2* regulates cambium development, we performed RNA-seq analysis of cambium cells isolated from the WT and *OE-PtrVCS2* to identify differentially expressed genes (DEGs) in response to *PtrVCS2* overexpression (Supplementary Table 2 and Methods). We identified 13,266 DEGs (FDR < 0.05) consisting of 6,653 upregulated and 6,613 downregulated ones. For VCS TF genes, 39 of the 95 VCS TF genes (Supplementary Table 1) were also downregulated, whereas 18 of these 95 TF genes were upregulated. These results suggest that *PtrVCS2* may function more as a trans-repressor for vascular cambium development, particularly in the cell proliferation system.

4. ChIP-sequencing of *OE-PtrVCS2*

To investigate which genes are directly regulated by PtrVCS2, we determined the global binding sites of PtrVCS2 by ChIP-seq analysis. We cloned *PtrVCS2* in-frame with a 3×FLAG tag sequence and overexpressed the resulting construct in *P. trichocarpa* under the control of a CaMV 35S promoter and identified binding sites using an anti-FLAG antibody in ChIP assays. We selected transgenic line #3 (*OE-PtrVCS2-3×FLAG #3*) showing the highest transgene transcript levels for further analysis (Extended Data Fig. 4a,b). Like the *OE-PtrVCS2* transgenics (Fig. 2c,d and Extended Data Fig. 1c,d), the *OE-PtrVCS2-3×FLAG #3* line had fewer cambium cell layers (Extended Data Fig. 4c,d). We carried out ChIP-seq analysis of cambium tissues from the *OE-PtrVCS2-3×FLAG #3* transgenic line and sequenced six ChIP-seq libraries (ChIP-DNA and input DNA, three biological replicates per library). We obtained 30.4~32.95 million uniquely aligned reads per library (Supplementary Table 3). The sequencing depth after removing duplication reads was 2.99×~3.33×, showing 60%~63% genome coverage (Supplementary Table 3). Data quality assessment using the irreproducible discovery rate framework with a 1% threshold indicated that the three replicates are highly reproducible (Supplementary Fig. 8a-i). Input DNA libraries were used as a control for ChIP-seq peak calling. We used a model-based analysis for ChIP-seq[18] to identify peaks and obtained 6,790 peaks for PtrVCS2 ($P <$ 1e-05 and conserved in at least two independent biological replicates, Extended Data Fig. 4e and Supplementary Table 3). Genes that contained one or more binding sites within 3 kb of the upstream putative promoter region were defined as target genes. Based on these criteria, we identified 2,087 putative PtrVCS2 target genes.

5. Protein-protein interaction motif feature of PtrVCS2

Phylogenetic and sequence analyses indicated that *PtrVCS2* encodes a putative zinc finger (ZF) protein (Extended Data Fig. 2a and Supplementary Fig. 9), is a homolog of Arabidopsis *MINI ZINC FINGER* (*MIF*) genes[12], and belongs to a subfamily of the zinc finger-homeodomain (ZF-HD) protein family[11,12,19]. The ZF-HD family members function in transcriptional regulation through the homeobox (HD) domain for DNA binding, and the zinc finger (ZF) domain for homo- and hetero-protein dimer formation[19-22]. PtrVCS2 harbors such ZF domain but lacks the HD domain (Supplementary Fig. 9), so we speculated that it targets genes by interacting with other HD-bearing TFs that can directly bind to such targets.

Supplementary Figures

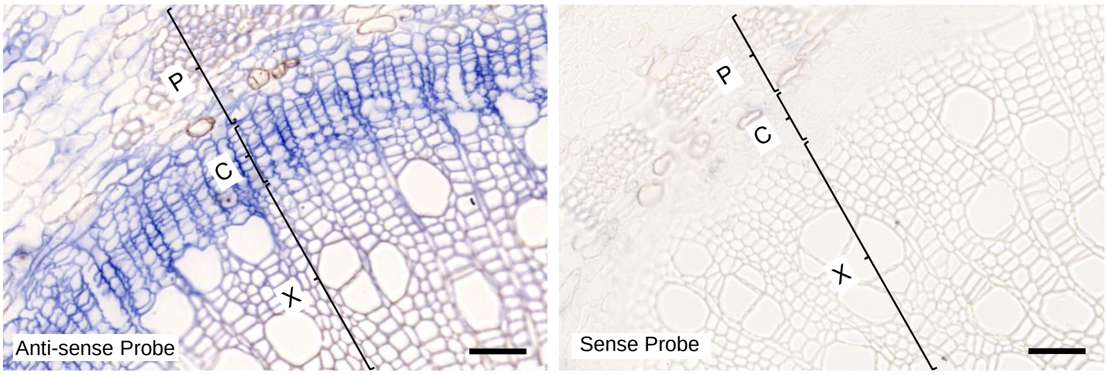

Supplementary Fig. 1 *In situ* hybridization of *PtrVCS2* mRNA. Hybridization with digoxigenin-labeled antisense RNA prob/es showed that *PtrVCS2* was preferentially expressed in the cambium zone. Paraffin sections are from the 6th internode of *P. trichocarpa* stems. Black brackets mark vascular cambium cells (C), phloem cells (P) and xylem cells (X). Scale bars, 50 µm. The experiments were repeated independently three times with similar results.

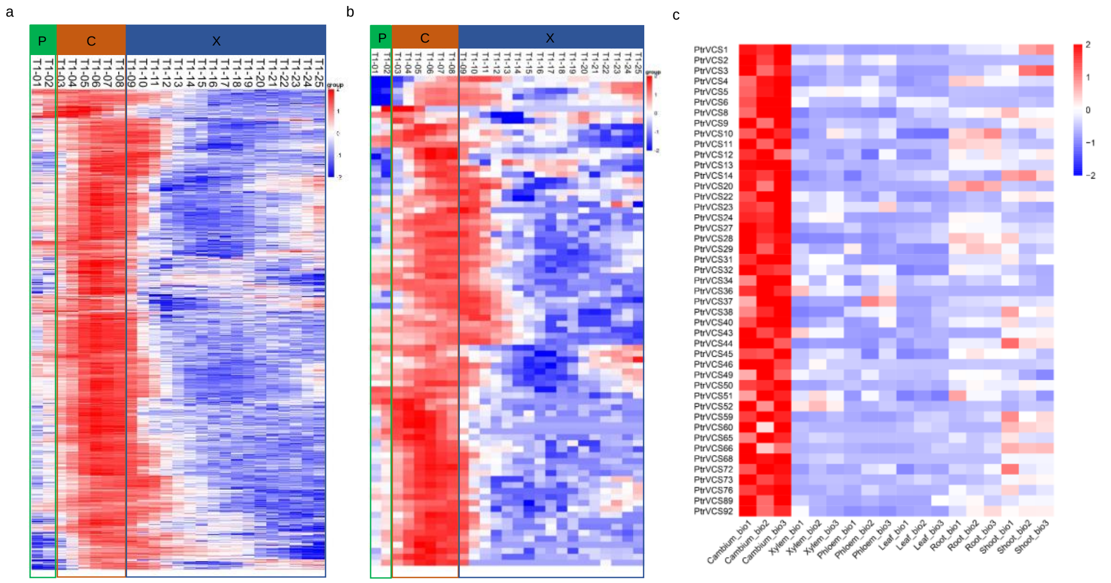

Supplementary Fig. 2 Expression profiles of vascular cambium-specific genes. a and **b**, Heat map showing the expression profiles of 1637 vascular cambium-specific (VCS) genes (**a**) and 82 VCS TF genes (**b**) across phloem (P), cambium (C) and xylem (X) tissues, based on the AspWood datasets[13] (http://aspwood.popgenie.org). **c**, Heat map showing the expression profiles of 45 VCS TF genes in cambium, xylem, and phloem cell types based on our LCM datasets and leaf, shoot (including shoot tips from the first to the third internode), and root tissues based on Shi's datasets[14]. In (**a**), (**b**), and (**c**), Expression values are scaled by row, with each row representing one gene. Expression values above the gene average are shown in red and below the average in blue, according to the color scale to the right of each heatmap.

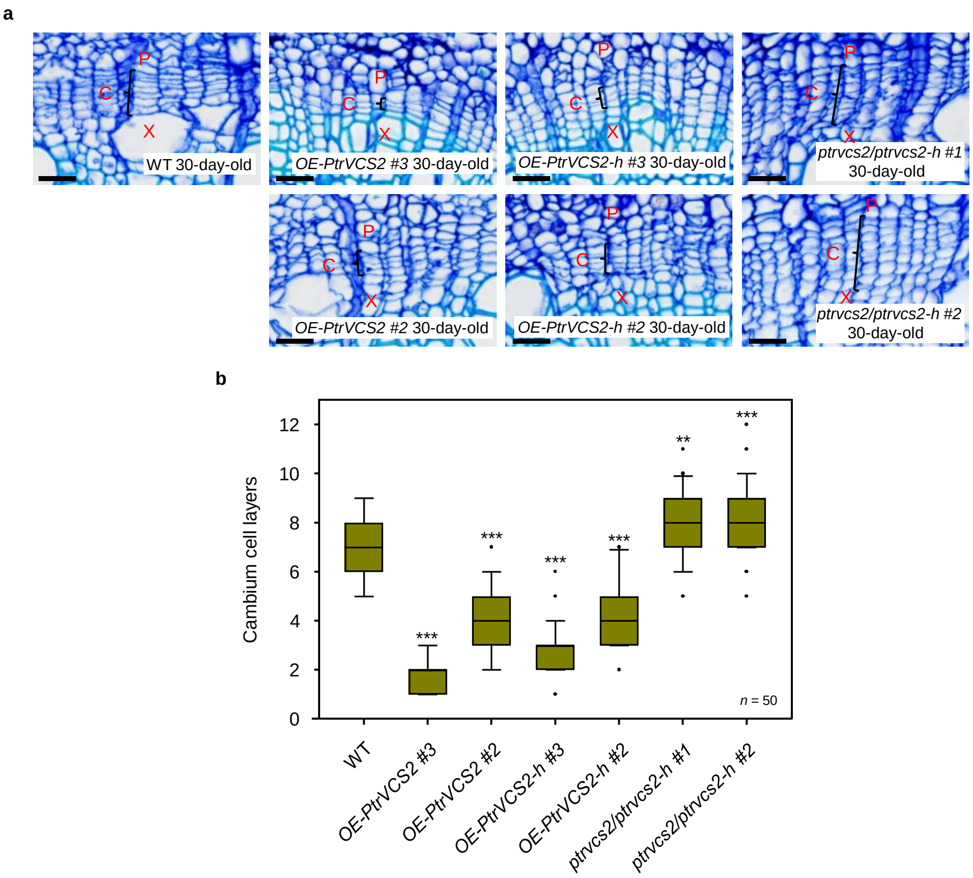

Supplementary Fig. 3 Cambium phenotype analysis of same-age internodes (30-day-old internodes) from WT, *OE-PtrVCS2*, *OE-PtrVCS2-h* transgenics and *ptrvcs2/ptrvcs2-h* mutants. a, Cross-sections of the same age internodes (30-day growth) from WT, *OE-PtrVCS2 #2*, *#3* transgenics, *OE-PtrVCS2-h #2*, *#3* transgenics, and *ptrvcs2/ptrvcs2-h #1*, *#2* mutants were stained with toluidine blue O. Black brackets mark the cambium cells in one radial cell file. Scale bars, 25 µm. C, cambium; P, phloem; X, xylem. **b**, Number of cambium cell layers in stem vascular tissues of WT, *OE-PtrVCS2 #2*, *#3* transgenics, *OE-PtrVCS2-h #2*, *#3* transgenics, and *ptrvcs2/ptrvcs2-h #1*, *#2* mutants of the same age internodes (30-day growth). Cambium cell layer numbers of at least ten radial cell files were counted within one cross-section from each biological replicate. Three biological replicates were carried

out. n=50. Boxes show the median with the upper and lower quantiles, and the whiskers represent the data range excluding outliers. Two-tailed Student's *t*-test: $^{**}P < 0.01$, $^{***}P < 0.001$. *P* values versus WT control for *OE-PtrVCS2 #3* is <0.0001, for *OE-PtrVCS2 #2* is <0.0001, *OE-PtrVCS2-h #3* is <0.0001, for *OE-PtrVCS2-h #2* is <0.0001, for *ptrvcs2/ptrvcs2-h #1* is 0.0089, for *ptrvcs2/ptrvcs2-h #2* is <0.0001.

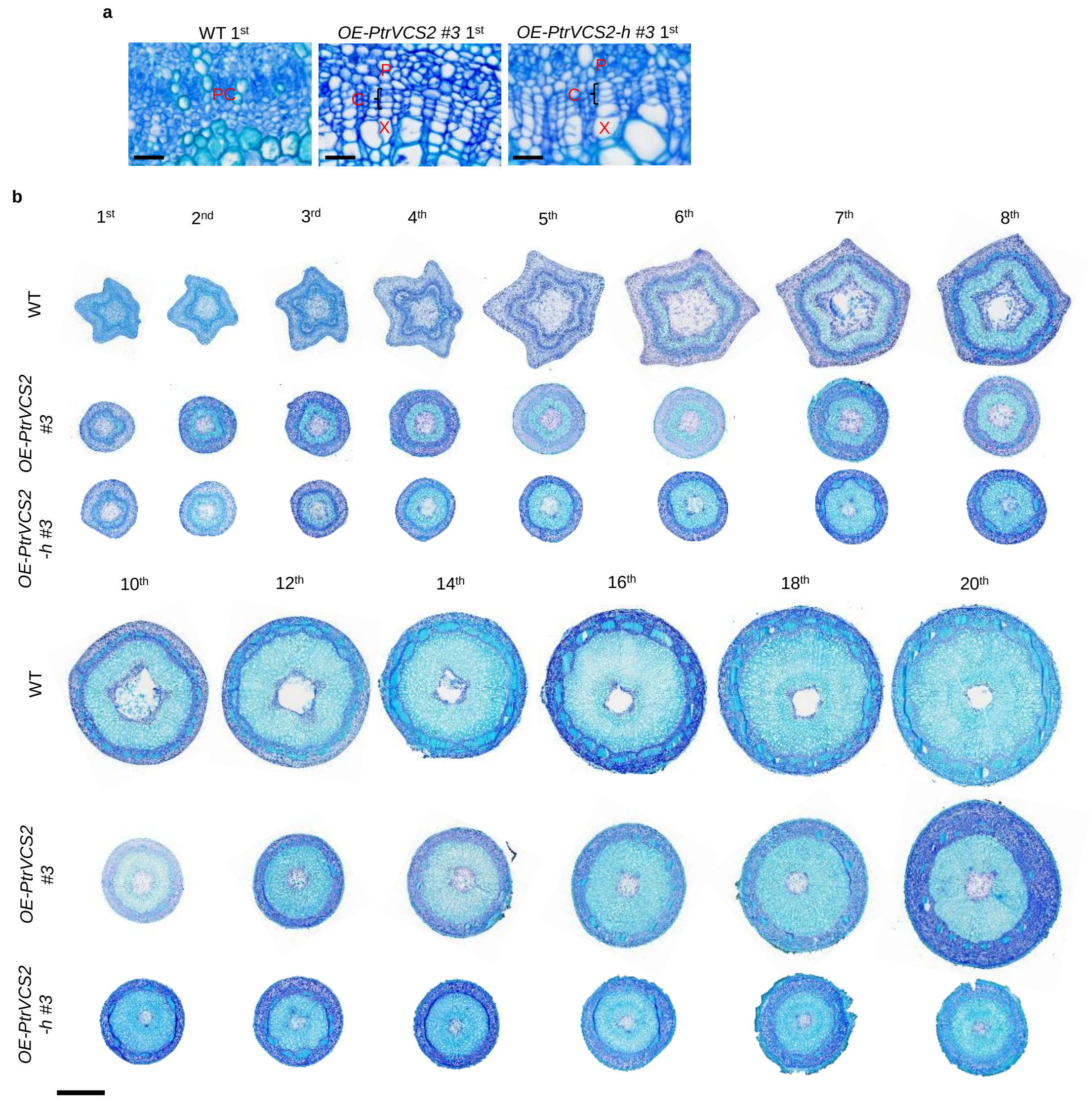

Supplementary Fig. 4 Histochemistry and histological analysis of internodes from WT, *OE-PtrVCS2*, and *OE-PtrVCS2-h* transgenics stained with toluidine blue O. Cross-sections of 14 internodes from the 1st (**a**) to the 20th (**b**) internodes of *P. trichocarpa* stems stained with toluidine blue O. Representative images from one biological replicate are shown, and the other two biological replicates were found with similar results. **a**, Black brackets mark the cambium cells in one radial cell file. Scale bars, 25 μm. PC, procambium; C, cambium; P, phloem; X, xylem. **b**, Scale bars,

1 mm.

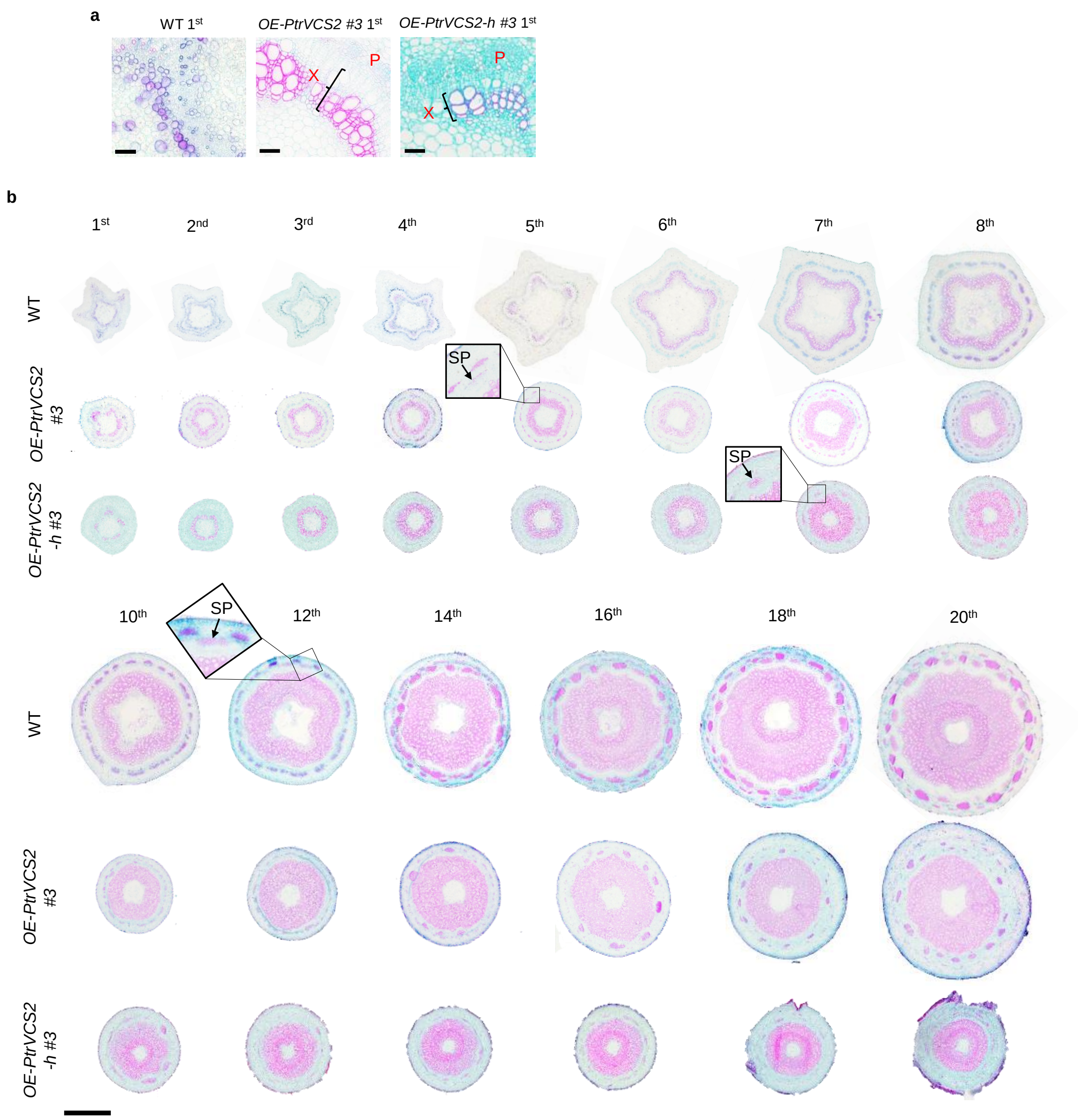

Supplementary Fig. 5 Histochemistry and histological analysis of internodes from WT, *OE-PtrVCS2*, and *OE-PtrVCS2-h* transgenics stained with safranin O and fast green. Cross-sections of 14 internodes from the 1st (**a**) to the 20th (**b**) of *P. trichocarpa* stems stained with safranin O and fast green. Representative images from one biological replicate are shown, and the other two biological replicates were found with similar results. **a**, Scale bars, 50 μm. X, xylem; P, phloem. **b**, Scale bars, 1 mm. SP, secondary phloem.

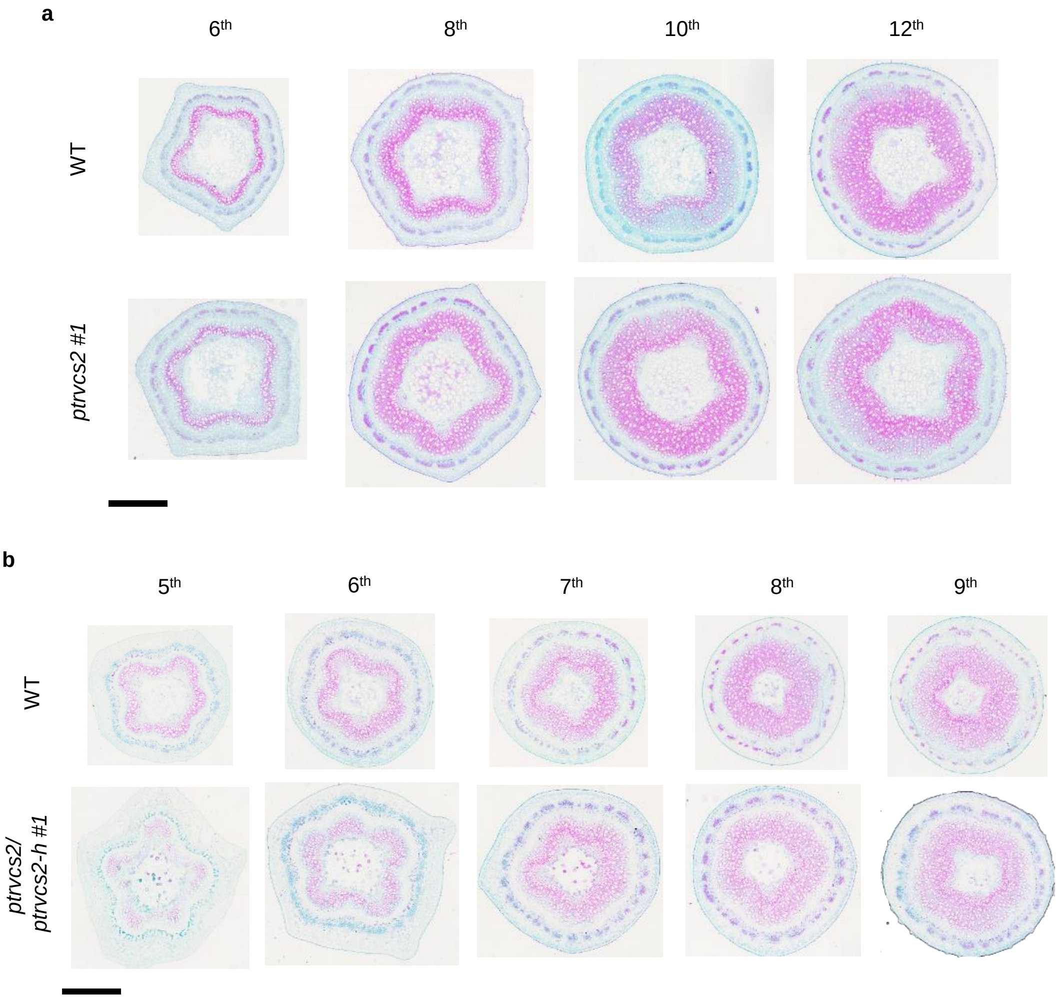

Supplementary Fig. 6 Histochemistry and histological analysis of internodes from WT, the *prtvcs2* single mutant (a) and the *ptrvcs2/ptrvcs2-h* double mutant (b). Cross-sections were stained with safranin O and fast green. Scale bars, 1 mm. Representative images from one biological replicate are shown, and the other two biological replicates were found with similar results.

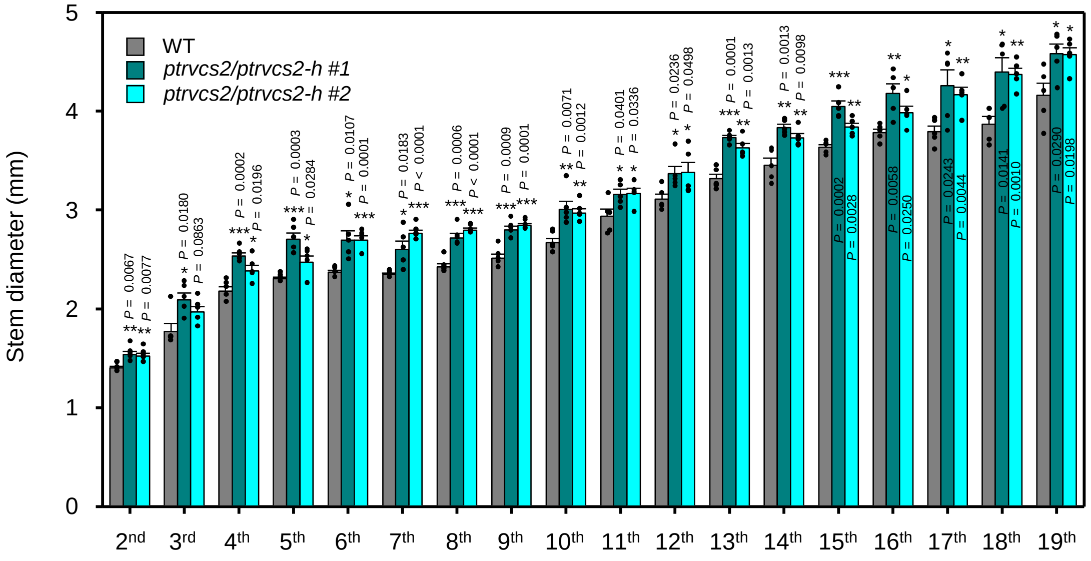

Supplementary Fig. 7 Stem diameter of wild-type and *ptrvcs2/ptrvcs2-h* double mutant plants. The data are shown as mean±s.e.m.; *n* =5 independent *P. trichocarpa* plants for each genotype. The asterisks indicate significant differences between the mutants and WT plants, as determined by two-tailed Student's *t*-test (**P* < 0.05, ***P* < 0.01, ****P* < 0.001).

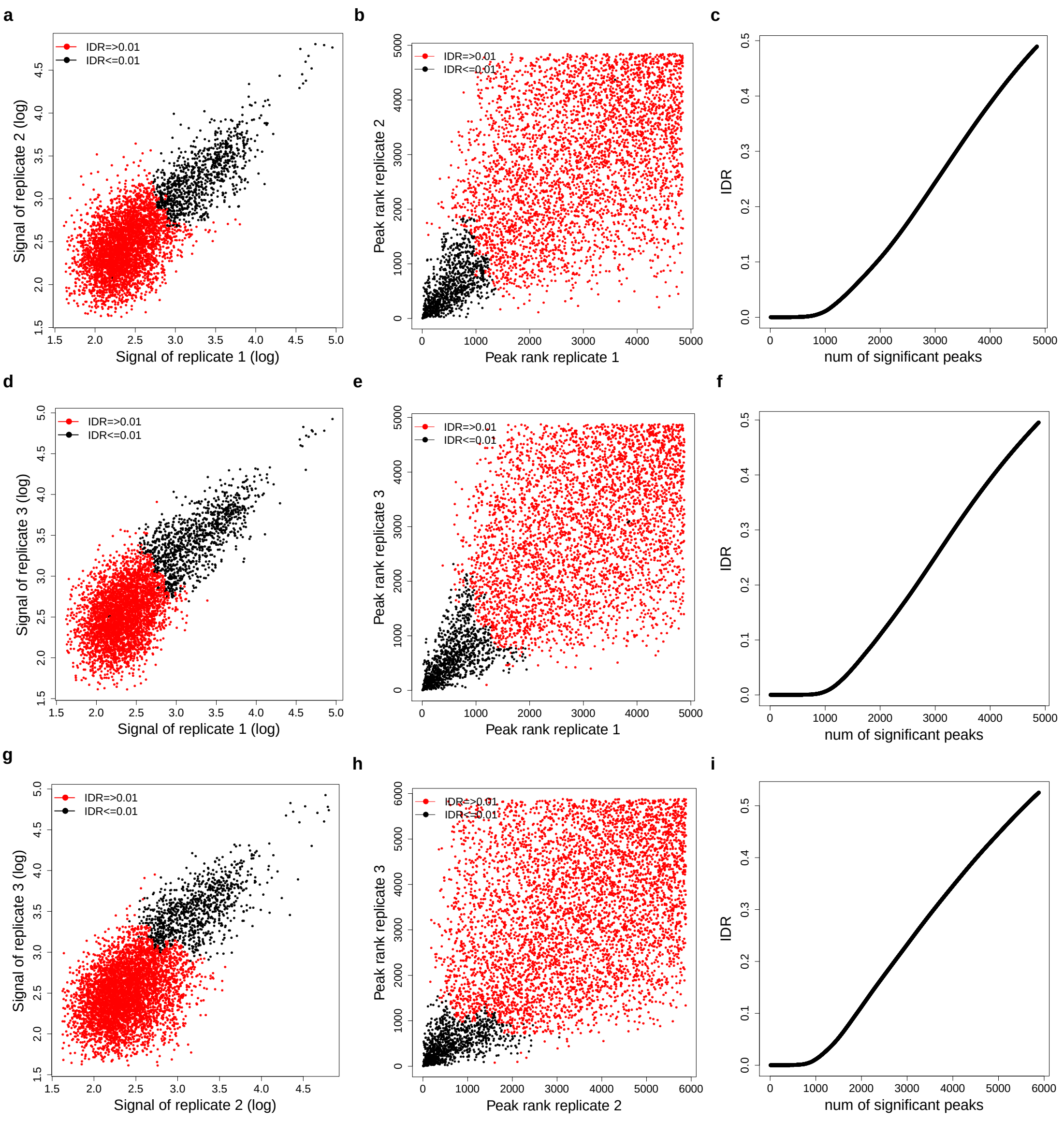

Supplementary Fig. 8 Irreproducible discovery rate (IDR) framework for assessing the reproducibility of PtrVCS2 ChIP-seq data sets. a, **d**, **g**, Scatter plots of signal scores of peaks that overlap in two replicates. **b**, **e**, **h**, Scatter plots of ranks of peaks that overlap in each pair of replicates. Note that low ranks correspond to high signal and vice versa. **c**, **f**, **i**, Estimated IDR as a function of different rank thresholds.

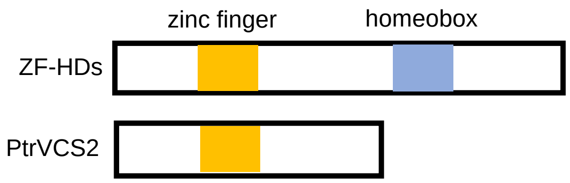

Supplementary Fig. 9 Schematic diagram of the functional domain in classical *P. trichocarpa* ZF-HD family proteins and PtrVCS2. PtrVCS2 has no homeobox domain, only the zinc finger domain.

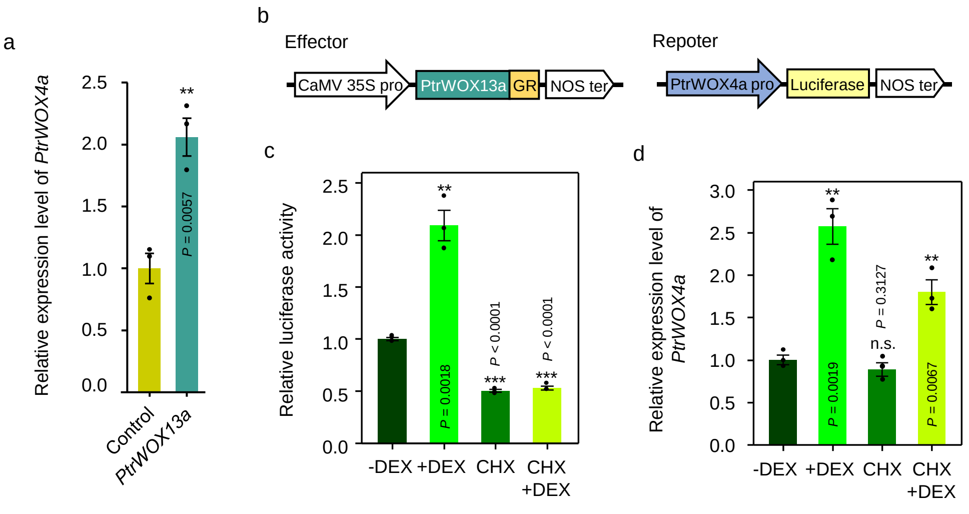

Supplementary Fig. 10 *PtrWOX13a* directly regulates *PtrWOX4a* transcription. a, Relative expression levels of *PtrWOX4a* in *P. trichocarpa* stem xylem protoplasts overexpressing *GFP* (Control) or *PtrWOX13a*. **b**, Schematic diagrams of the effector and reporter constructs used in the glucocorticoid receptor-based inducible gene expression assays[23]. **c**, Luciferase activities in *P. trichocarpa* stem xylem protoplasts co-transfected with the effector and reporter constructs. Luciferase activity was induced by adding dexamethasone (DEX) and was completely abolished by treating with the protein synthesis inhibitor cycloheximide (CHX). **d**, Relative expression levels of *PtrWOX4a* in *P. trichocarpa* stem xylem protoplasts expressing the effector construct. The *PtrWOX4a* transcription was activated by DEX even in the presence of CHX, indicating that PtrWOX13a directly regulates the *PtrWOX4a* expression. In (**a**), (**c**), and (**d**), control samples (no DEX) were set to 1. The data are shown as mean±s.e.m.; n =3 biological replicates (three independent batches of stem xylem protoplasts transfections; Two-tailed Student's t-test: $^{**}P < 0.01$, $^{***}P < 0.001$; n.s., no significant difference).

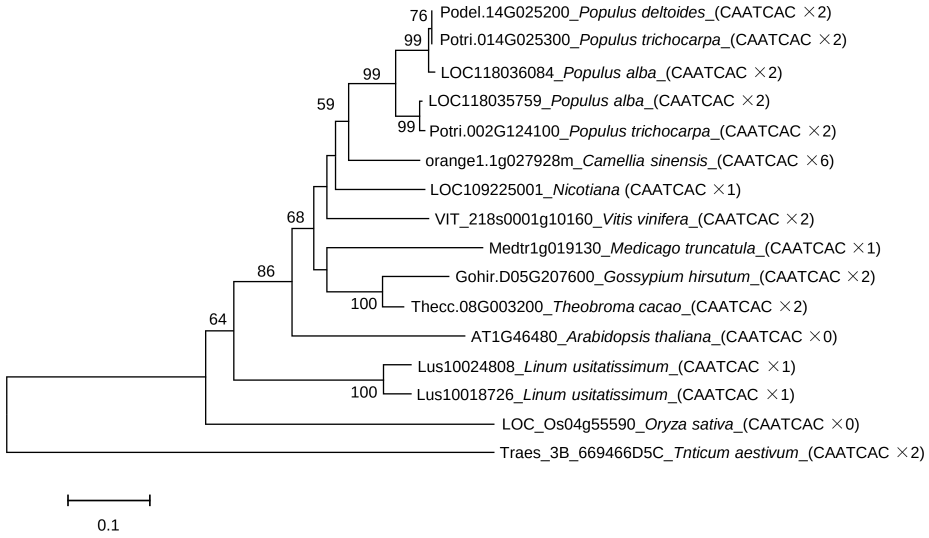

Supplementary Fig. 11 Phylogenetic tree of WOX4 proteins from 13 species. The number of the CAATCAC motif in the 3-kb promoter region of *WOX4* genes is shown in the panel. The phylogenetic tree was constructed using MEGA 5 with the neighbor-joining method and 1000 bootstrap replicates. Bar, 0.1 changes per amino acid position.

Supplementary Information References

1. Zhang, J. et al. Transcriptional regulatory framework for vascular cambium development in Arabidopsis roots. *Nat. Plants* **5**, 1033-1042 (2019).
2. Zhu, Y., Song, D., Sun, J., Wang, X. & Li, L. PtrHB7, a class III HD-Zip gene, plays a critical role in regulation of vascular cambium differentiation in *Populus*. *Mol. Plant* **6**, 1331-1343 (2013).
3. Zhu, Y., Song, D., Xu, P., Sun, J. & Li, L. A HD-ZIP III Gene, PtrHB4, Is Required for Interfascicular Cambium Development in *Populus*. *Plant Biotechnol. J.* **16**, 808-817 (2018).
4. Robischon, M., Du, J., Miura, E. & Groover, A. The *Populus* class III HD ZIP, popREVOLUTA, influences cambium initiation and patterning of woody stems. *Plant Physiol.* **155**, 1214-1225 (2011).
5. Smit, M. E. et al. A PXY-Mediated Transcriptional Network Integrates Signaling Mechanisms to Control Vascular Development in *Arabidopsis*. *Plant Cell* **32**, 319-335 (2020).
6. Kucukoglu, M., Nilsson, J., Zheng, B., Chaabouni, S. & Nilsson, O. WUSCHEL-RELATED HOMEOBOX4 (WOX4)-like genes regulate cambial cell division activity and secondary growth in Populus trees. *New Phytol.* **215**, 642-657 (2017).
7. Hirakawa, Y., Kondo, Y. & Fukuda, H. TDIF peptide signaling regulates vascular stem cell proliferation via the WOX4 homeobox gene in *Arabidopsis*. *Plant Cell* **22**, 2618-2629 (2010).
8. Suer, S., Agusti, J., Sanchez, P., Schwarz, M. & Greb, T. WOX4 imparts auxin responsiveness to cambium cells in *Arabidopsis*. *Plant Cell* **23**, 3247-3259 (2011).

9. Mackay, J. P. & Crossley, M. Zinc fingers are sticking together. *Trends Biochem. Sci.* **23**, 1-4 (1998).
10. Takatsuji, H. Zinc-finger proteins: the classical zinc finger emerges in contemporary plant science. *Plant Mol. Biol.* **39,** 1073-1078 (1999).
11. Liu, M. et al. Genome-wide investigation of the ZF-HD gene family in Tartary buckwheat (Fagopyrum tataricum). *BMC Plant Biol.* **19**, 248 (2019).
12. Hu, W. & Ma, H. Characterization of a novel putative zinc finger gene MIF1: involvement in multiple hormonal regulation of *Arabidopsis* development. *Plant J.* **45**, 399-422 (2006).
13. Sundell, D. et al. AspWood: High-spatial-resolution transcriptome profiles reveal uncharacterized modularity of wood formation in *Populus tremula*. *Plant Cell* **29**, 1585-1604 (2017).
14. Shi, R. et al. Tissue and cell-type co-expression networks of transcription factors and wood component genes in Populus trichocarpa. *Planta* **245**, 927-938 (2017).
15. Li, L. et al. The last step of syringyl monolignol biosynthesis in angiosperm dicots is regulated by a novel gene encoding sinapyl alcohol dehydrogenase. *Plant Cell* **13**, 1567-1586 (2001).
16. Esau, K. Vascular Differentiation in Plants (New York: Holt, Rinehart and Winston, 1965).
17. Evert, R. F. & Eichhorn, S. E. Esau's Plant Anatomy: Meristems, Cells, and Tissues of the Plant Body: Their Structure, Function, and Development, Ed 3 (Hoboken, NJ: John Wiley & Sons, 2006).
18. Zhang, Y. et al. Model-based analysis of ChIP-Seq (MACS). *Genome Biol.* **9**,

R137 (2008).

19. Hu, W., dePamphilis, C. W & Ma, H. Phylogenetic analysis of the plant-specific zinc finger-homeobox and mini zinc finger gene families. *J. Integr. Plant Biol.* **50**, 1031-1045 (2008).

20. Windhövel, A., Hein, I., Dabrowa, R. & Stockhaus, J. Characterization of a novel class of plant homeodomain proteins that bind to the C4 phosphoenolpyruvate carboxylase gene of Flaveria trinervia. *Plant Mol. Biol.* **45**, 201-214 (2001).

21. Park, H. C. et al. Pathogen-induced binding of the soybean zinc finger homeodomain proteins GmZF-HD1 and GmZF-HD2 to two repeats of ATTA homeodomain binding site in the calmodulin isoform 4 (GmCaM4) promoter. *Nucleic acids res.* **35**, 3612-3623 (2007).

22. Perrella, G. et al. ZINC-FINGER interactions mediate transcriptional regulation of hypocotyl growth in Arabidopsis. *Proc. Natl. Acad. Sci. USA.* **115**, E4503-E4511 (2018).

23. Aoyama, T. & Chua, N.H. A glucocorticoid-mediated transcriptional induction system in transgenic plants. *Plant J.* **11**, 605-612 (1997)
