# Supplementary material for: Cell-type-specific PtrWOX4a and PtrVCS2 form a regulatory nexus with a histone modification system for stem cambium development in Populus trichocarpa
Source: Nat Plants. 2023 Jan 9;9(1):96–111. doi: 10.1038/s41477-022-01315-7 (PMC9873556; doi:10.1038/s41477-022-01315-7)
Supplement: Supplementary file 2 — Reporting Summary [file 41477_2022_1315_MOESM2_ESM.pdf]

nature portfolio

Corresponding author(s): Wei Li

Last updated by author(s): Nov 12, 2022

Reporting Summary

Nature Portfolio wishes to improve the reproducibility of the work that we publish. This form provides structure for consistency and transparency in reporting. For further information on Nature Portfolio policies, see our Editorial Policies and the Editorial Policy Checklist.

Statistics

For all statistical analyses, confirm that the following items are present in the figure legend, table legend, main text, or Methods section.

n/a	Confirmed	
☐	☒	The exact sample size (n) for each experimental group/condition, given as a discrete number and unit of measurement
☐	☒	A statement on whether measurements were taken from distinct samples or whether the same sample was measured repeatedly
☐	☒	The statistical test(s) used AND whether they are one- or two-sided<br>*Only common tests should be described solely by name; describe more complex techniques in the Methods section.*
☒	☐	A description of all covariates tested
☒	☐	A description of any assumptions or corrections, such as tests of normality and adjustment for multiple comparisons
☐	☒	A full description of the statistical parameters including central tendency (e.g. means) or other basic estimates (e.g. regression coefficient) AND variation (e.g. standard deviation) or associated estimates of uncertainty (e.g. confidence intervals)
☐	☒	For null hypothesis testing, the test statistic (e.g. F, t, r) with confidence intervals, effect sizes, degrees of freedom and P value noted<br>*Give P values as exact values whenever suitable.*
☒	☐	For Bayesian analysis, information on the choice of priors and Markov chain Monte Carlo settings
☒	☐	For hierarchical and complex designs, identification of the appropriate level for tests and full reporting of outcomes
☒	☐	Estimates of effect sizes (e.g. Cohen's d, Pearson's r), indicating how they were calculated

Our web collection on statistics for biologists contains articles on many of the points above.

Software and code

Policy information about availability of computer code

Data collection	High-throughput sequencing: Illumina platform<br>Microscopy images for histochemical and histological analysis and images collection for RNA in situ hybridization: scanner M8 (Precipoint)<br>Reverse transcription-quantitative PCR data: Agilent M×3000P Real-Time PCR System<br>Confocal images: Zeiss LSM 800<br>Histone acetyltransferase and luciferase activity data: BioTek Synergy H1
Data analysis	RNA-seq analysis: SOAPnuke, Bowtie2 (v2.2.5), DESeq2 (v1.4.5)<br>ChIP-seq analysis: FASTX-Toolkit (v0.0.14), Bowtie 2 (v2.3.5.1), MACS2, MEME-ChIP<br>Statistical analyses (mean, standard error of the mean, and Student's t-test): Microsoft Excel 2019<br>Phylogenetic analysis: MEGA 5<br>Fluorescence signals measurement: ImageJ (1.53e)<br>Protein sequence alignment: Clustal W<br>sgRNAs analysis: CRISPR-P 2.0<br>Other: SigmaPlot 10<br>No custom code was used to analyse the data in this study.

For manuscripts utilizing custom algorithms or software that are central to the research but not yet described in published literature, software must be made available to editors and reviewers. We strongly encourage code deposition in a community repository (e.g. GitHub). See the Nature Portfolio guidelines for submitting code & software for further information.

Data

Policy information about availability of data

All manuscripts must include a data availability statement. This statement should provide the following information, where applicable:

- Accession codes, unique identifiers, or web links for publicly available datasets
- A description of any restrictions on data availability
- For clinical datasets or third party data, please ensure that the statement adheres to our policy

The RNA-seq and ChIP-seq raw sequencing data have been deposited in the National Center for Biotechnology Information Sequence Read Archive under accession number SRR18274403-SRR18274417 and SRR18272729-SRR18272734. The ChIP-seq analyzed data have been deposited in the Gene Expression Omnibus database under accession number GSE201005. Sequence data from this article can be found in P. trichocarpa genome v3.0 (Phytozome, https://phytozome.jgi.doe.gov/pz/portal.html) under the accession numbers listed in Supplementary Table 1 and Supplementary Table 6. The data supporting the findings of this study are available within the article and its Supplementary Information files.

Field-specific reporting

Please select the one below that is the best fit for your research. If you are not sure, read the appropriate sections before making your selection.

☒ Life sciences ☐ Behavioural & social sciences ☐ Ecological, evolutionary & environmental sciences

For a reference copy of the document with all sections, see nature.com/documents/nr-reporting-summary-flat.pdf

Life sciences study design

All studies must disclose on these points even when the disclosure is negative.

Sample size	Appropriate sample size has been mentioned in the figure legends of respective experiments and described in the methods. The sample size was determined based on common empirical knowledge in similar scientific studies and the feasibility of sample collection. The sample size was enough to result in statistical significance and reproducibility.
Data exclusions	No data was excluded.
Replication	The number of replication is indicated in the figure legends of respective experiments and described in the methods. All the experiments were repeated at least three times independently with similar results.
Randomization	Different genotypes were grown in individual pots and were allocated randomly in a walk-in growth chamber.
Blinding	All the experiments were carried out without prior knowledge of the final outcome, and blinding was not applicable to the study.

Reporting for specific materials, systems and methods

We require information from authors about some types of materials, experimental systems and methods used in many studies. Here, indicate whether each material, system or method listed is relevant to your study. If you are not sure if a list item applies to your research, read the appropriate section before selecting a response.

Materials & experimental systems

n/a	Involved in the study	
☐	☒	Antibodies
☒	☐	Eukaryotic cell lines
☒	☐	Palaeontology and archaeology
☒	☐	Animals and other organisms
☒	☐	Human research participants
☒	☐	Clinical data
☒	☐	Dual use research of concern

Methods

n/a	Involved in the study	
☐	☒	ChIP-seq
☒	☐	Flow cytometry
☒	☐	MRI-based neuroimaging

Antibodies

Antibodies used	Anti-FLAG antibody, Sigma, Cat# F1804<br>Anti-Histone H3 (acetyl K9) antibody, Abcam, Cat# ab10812<br>Anti-Histone H3 (acetyl K14) antibody, Abcam, Cat# ab52946<br>Anti-Histone H3 (acetyl K27) antibody, Abcam, Cat# ab4729<br>Anti-PtrVCS2 antibody, Abmart, custom made<br>Anti-IgG antibody, Abcam, Cat# ab205719

	Anti-His antibody, Abcam, Cat# ab1187<br>Anti-S antibody, Abcam, Cat# ab183674
Validation	Validations are based on the datasheet from the manufacturer and the detailed information is as follows.<br>Anti-FLAG antibody (https://www.sigmaaldrich.cn/CN/en/product/sigma/f1804)<br>Anti-Histone H3 (acetyl K9) antibody (https://www.abcam.com/histone-h3-acetyl-k9-antibody-chip-grade-ab10812.html)<br>Anti-Histone H3 (acetyl K14) antibody (https://www.abcam.com/histone-h3-acetyl-k14-antibody-ep964y-chip-grade-ab52946.html)<br>Anti-Histone H3 (acetyl K27) antibody (https://www.abcam.com/histone-h3-acetyl-k27-antibody-chip-grade-ab4729.html)<br>Anti-IgG antibody (https://www.abcam.cn/Goat-Mouse-IgG-HL-HRP-ab205719.html)<br>Anti-His antibody (https://www.abcam.cn/hrp-6x-his-tag-antibody-ab1187.html)<br>Anti-S antibody (https://www.abcam.cn/s-tag-antibody-ab183674.html)

ChIP-seq

Data deposition

☒ Confirm that both raw and final processed data have been deposited in a public database such as GEO.

☒ Confirm that you have deposited or provided access to graph files (e.g. BED files) for the called peaks.

Data access links<br>*May remain private before publication.*	The raw sequencing data of ChIP-seq have been deposited in the National Center for Biotechnology Information Sequence Read Archive under accession number SRR18274403-SRR18274408. The analyzed data of ChIP-seq have been deposited in the Gene Expression Omnibus database under accession number GSE201005.
Files in database submission	SRR18274407 bio1 IP<br>SRR18274408 bio1 input<br>SRR18274405 bio2 IP<br>SRR18274406 bio2 input<br>SRR18274403 bio3 IP<br>SRR18274404 bio3 input<br>bio1IP.bw<br>bio2IP.bw<br>bio3IP.bw<br>bioinput.bw<br>bio1_IPvspoolinput_peaks.narrowPeak<br>bio2_IPvspoolinput_peaks.narrowPeak<br>bio3_IPvspoolinput_peaks.narrowPeak<br>bio1IP_raw.R1.fq.gz<br>bio2IP_raw.R1.fq.gz<br>bio3IP_raw.R1.fq.gz<br>bio1input_raw.R1.fq.gz<br>bio2input_raw.R1.fq.gz<br>bio3input_raw.R1.fq.gz
Genome browser session<br>(e.g. UCSC)	A link to an anonymized genome browser session is not available now.

Methodology

Replicates	3 biological replicates
Sequencing depth	All ChIP-seq samples were 50-bp single-end reads sequencing.<br>Sample name; total reads; uniquely mapped reads:<br>bio1 IP; 63321007; 31652206<br>bio1 input; 64747205; 32037559<br>bio2 IP; 65575403; 30396484<br>bio2 input; 62353145; 30862321<br>bio3 IP; 63975664; 32950039<br>bio3 input; 62184627; 30730123
Antibodies	Anti-FLAG antibody, Monoclonal produced in mouse, Sigma, Cat# F1804
Peak calling parameters	The raw sequencing reads were processed to trim adaptor sequences (fastx_clipper –a CCTTAAGG) and filter low-quality reads (Fastq_quality_filter –p 85 –q 20) using FASTX-Toolkit (v0.0.14). The processed reads were mapped to Populus trichocarpa genome reference v3.0 using Bowtie 2 (v2.3.5.1) with up to 1 mismatch allowed (Bowtie2 -N 1). Only uniquely mapped reads with removing duplicated reads were used for peak identification. Default parameters of MACS2 were used for peak calling with p-value < 1e-05 in this study.
Data quality	Most of the reads (>85%) generated from the experiment were mapped to the the Populus trichocarpa genome v3.0. Only uniquely mapped reads with removing duplicated reads were used for peak identification. Peaks identified in at least two biological replicates (peaks summits between replicates were less than 100 bp) were defined as common peaks. Over 50% peaks of each biological replicate were identified as common peaks. Common peaks between biological replicates were merged and assigned to the closest genes. Data quality assessment using the irreproducible discovery rate framework with a 1% threshold indicated that the three replicates are highly reproducible (shown in supplementary figure 8).

Software	FASTX-Toolkit (v0.0.14), Bowtie 2 (v2.3.5.1), MACS2
